# Start, Stop, Rewind, Repeat—Cyclic Exposure of Adipose Stromal Cells‐derived Cartilage Organoids to Chondrogenic and Proliferative Cues to Achieve Scaled‐up and Customizable Bone Formation by Endochondral Ossification

**DOI:** 10.1002/adhm.202504880

**Published:** 2026-01-09

**Authors:** Pablo Pfister, Emilien Lhospice, Andrés García‐García, Robert Paillaud, Sebastian Jung, Romain Schaller, Elisabeth A. Kappos, Claude Jaquiéry, Tarek Ismail, Dirk J. Schaefer, Michael de Wild, Ivan Martin, Alexandre Kaempfen, Arnaud Scherberich, Adrien Moya

**Affiliations:** ^1^ Department of Biomedicine University of Basel University Hospital of Basel Basel Switzerland; ^2^ Department of Plastic Reconstructive Aesthetic and Hand Surgery University Hospital Basel Basel Switzerland; ^3^ School of Life Sciences HLS Institute For Medical Engineering and Medical Informatics IM2 University of Applied Sciences Northwestern Switzerland FHNW Muttenz Switzerland; ^4^ Department of Oral and Maxillofacial Surgery University Hospital Basel Basel Switzerland

**Keywords:** adipose‐derived stromal cells, bone tissue engineering, cartilage organoids, clinical translation, cyclic bottom‐up approach, endochondral ossification

## Abstract

Developmental tissue engineering (TE) strategies recapitulating endochondral ossification (ECO)—the major ossification pathway in bone development and repair– are a promising avenue for the treatment of critical size and congenital bone defects. In this work, we develop a customizable approach using adipose stromal cells (ASC)‐derived cartilage organoids as building blocks to generate clinically relevant grafts. Our hypothesis is that progenitor cells rather than mature chondrocytes would enable robust cartilage organoids fusion allowing graft tunability and scaling up. Using a cyclic approach alternating chondrogenic and proliferative cues we produce cartilage organoids surrounded by stromal chondrogenic progenitors. When re‐exposed to chondrogenic medium, this perichondrial layer forms new cartilage tissue and acts as a biological cement in between cartilage organoids. A key feature of our approach is the iterative aspect of the protocol, where scaled up cartilage tissues obtained can themselves be used as building blocks to create larger tissue. Finally, in vivo, these grafts remodel efficiently into functional and mechanically apt bone organs mirroring ECO during skeletal development over the course of 24 weeks. Collectively, our findings provide a strong proof of concept of the envisioned TE strategy paving the way for a clinical application in the near future.

## Graphical abstract

This study exploits the plasticity of ASCs‐derived cartilage organoids which generate a perichondrial layer of MSCs when exposed to cyclic chondrogenic/proliferative cues. Using these organoids as building blocks, we develop (i) Phalange Shaped Tissue Engineered Cartilage (Pa‐TECs), recapitulating endochondral ossification suitable for the treatment of children suffering from symbrachydactyly and (ii) larger and more complex grafts are produced by repeating the cycles.

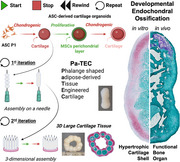

## 1 Introduction

Despite remarkable regenerative capacities [1, 2, 3], bone tissue is the second most transplanted tissue after blood transfusion with over two million procedures performed annually worldwide [4]. In addition, patients receiving bone grafts can suffer from long‐term invalidity which are associated with high socio‐economic impact. Autologous bone graft—bone harvested from the patient own's body— remains the gold standard for the treatment of critical size bone defects, tumor resection and congenital bone deformities [5, 6]. However, the severe donor site morbidity and limited tissue availability associated with autologous bone grafting has prompted for development of tissue engineering (TE) approaches to generate clinical alternatives [6, 7]. While most of the bone TE strategies are based on the intramembranous ossification (IMO) pathway, a change in paradigm was proposed with new “developmental engineering” approaches focusing on recapitulating the endochondral ossification (ECO) in the years 2009–2010 [8, 9].

During ECO—the major ossification pathway for the development and repair of long bones and bones of the hand and feet—mesenchymal stromal cells (MSCs) first condense and form an intermediate avascular cartilage anlage which upon vascularization remodels into a bone organ [10, 11]. Compared to IMO, ECO based approaches appear more suited for the generation of clinically relevant bone grafts. First, hypertrophic chondrocytes are more capable than progenitor cells to withstand the harsh avascular environment of large bone defects because it resembles their physiological environment. Second, hypertrophic chondrocytes actively promote angiogenesis by secreting VEGF thus favoring host‐recruited vascularization post‐implantation [10, 12, 13]. Several research groups, including our own, have sought and succeeded in generating cartilage or hypertrophic cartilage tissues—now termed cartilage organoids—from various cell sources in vitro that faithfully recapitulate ECO in vivo [9, 14, 15, 16, 17, 18, 19, 20, 21]. In particular, our group has shown that, despite their non‐skeletal origin adipose‐derived stromal cells (ASCs) were capable of generating bone organ when adequately primed in vitro [22]. However, despite an intense research focus on bone and cartilage organoids in the last 15 years [23, 24], these organoids have not yet been used in the clinic to treat bone defects, mainly due to the rather limited size of their resulting bone organs.

Recent advances in hydrogels functionalization, scaffold tunability, and 3D printing have been shown to improve ECO with increasing control over the shape and size [25, 26, 27, 28, 29, 30, 31]. Alternatively, bottom‐up approaches—consisting in aggregate of microtissues/organoids termed assembloids—are becoming more refined and can better replicate complex tissue architectures leading to robust ECO [32, 33, 34, 35]. These approaches could be integrated in the future in a fully automated and standardized biomanufacturing pipeline [36]. Sophisticated strategies using a combination of 3D printing with bioinks containing organoids are also being explored. For example, *Zhang* et al. report the generation of ossification center‐like organoids which promoted rapid bone healing in a rat calvaria defect model through the recruitment of *Krt8^+^* skeletal stem cells (SSCs) using a combination of osteogenic bone marrow MSCs derived spheroids and neurotrophic and proangiogenic factors [37]. Recently, an impressive study by *Xiong* et al. describes chondro‐ and osteo‐spheroids obtained from *Sox9^+^* sclerotomal progenitors derived from induced pluripotent stem cells (iPSCs) that displayed self‐organized growth plate‐like structures which closely resemble primary growth plates at the molecular and cellular levels and enabled tissue growth in vivo [38]. Collectively, these innovations enable an efficient ECO recapitulation by closely mimicking the tissue development, growth factors gradients and optimizing the cellular response.

Envisioned clinical TE strategies should be kept reasonably simple yet robust to ensure both efficacy and feasibility. In this context, ASCs obtained from the stromal vascular fraction (SVF) of lipoaspirate or adipose tissue is an attractive cell source given their abundance, availability and capacity to recapitulate the process of ECO [39, 40]. Recently, our group could demonstrate that the maturation level of adipose‐derived cartilage organoids was predictive of their in vivo bone formation [22]. By modulating the size and composition of collagen sponge scaffold we showed that it was possible to increase the size of hypertrophic cartilage grafts (up to 390 mm^3^) [41]. However, the resulting cartilage formation was heterogenous leading to partial and reduced bone remodeling which ultimately prompted us to develop a new strategy for scaling up.

Here, we investigated a bottom‐up approach using ASCs‐derived cartilage organoids as biological building blocks to generate large TE cartilage grafts. We hypothesized that progenitor cells rather than mature chondrocytes would facilitate organoids assembly and ensure tissue integrity. We thus tested a novel cyclic chondrogenic and proliferative protocol that replenishes the chondrogenic progenitor pool thereby forming an undifferentiated perichondrial layer surrounding cartilage organoids. We targeted cartilage organoid fusion utilizing perichondrial layers as a biological cement to enable the production of large and customizable cartilage tissues in vitro. Finally, we established proof of feasibility and efficacy in a pre‐clinical animal model of our proposed paradigm. Specifically, we generated phalange‐shaped hypertrophic cartilage grafts and validated their capacity to robustly recapitulate ECO in an ectopic immuno‐compromised mice model mimicking the scenario of an autologous TE approach. This innovative developmental strategy, suitably embedded within good manufacturing practice (GMP) settings and advanced therapy medicinal product (ATMP) regulations, could be implemented to treat congenital bone defect in a clinical pediatric setting.

## 2 Results and Discussion

### 2.1 A novel cyclic in vitro Approach to Replenish Chondrogenic Progenitors in ASCs Derived Cartilage Organoids

**FIGURE 1:**
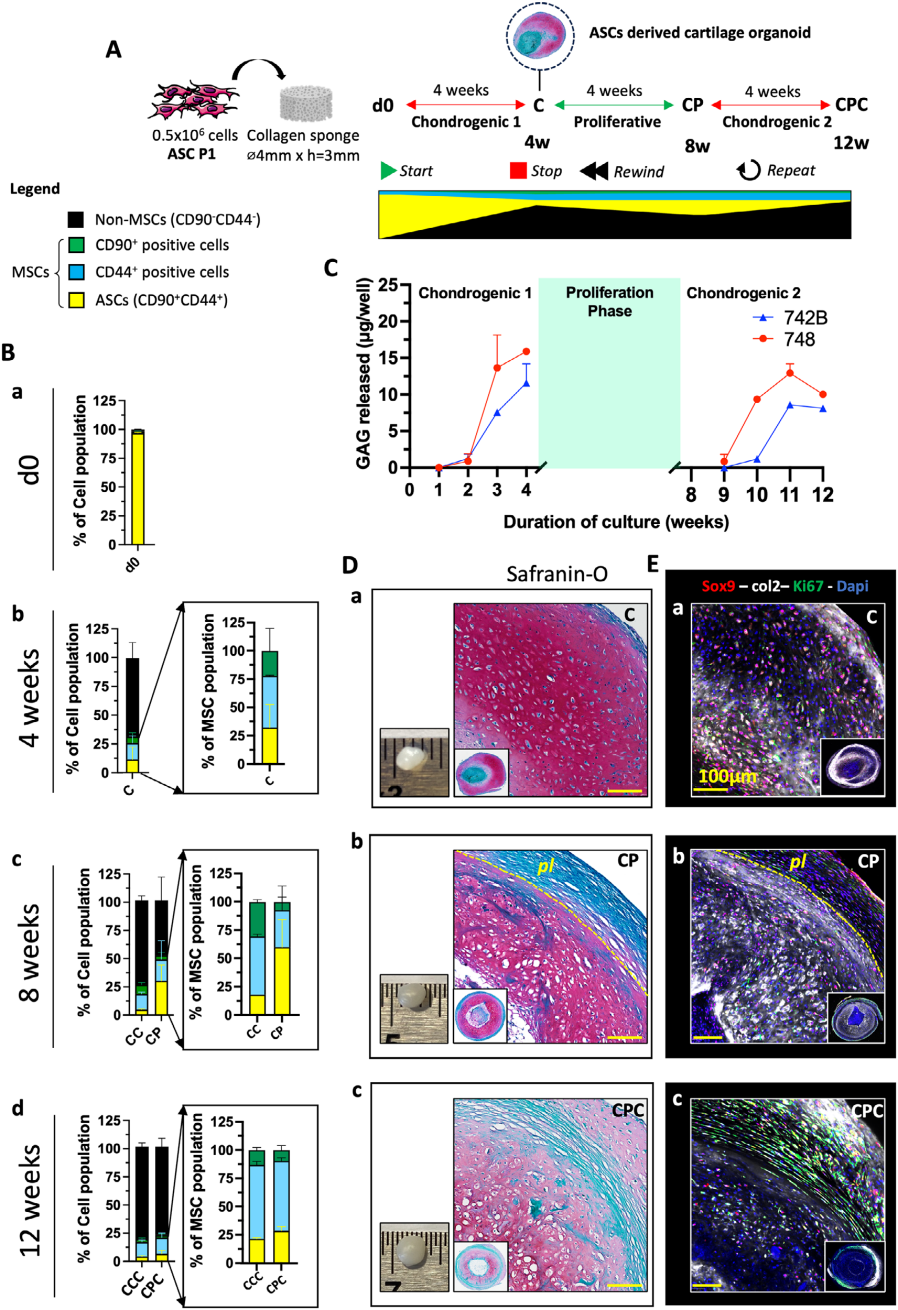
ASCs derived cartilage organoids respond to proliferative cues generating a perichondrial layer of chondrogenic progenitors. ((0.5 × 10^6^ ASCs p1 were seeded on collagen sponge scaffold (d0) and exposed to chondrogenic media for 4 weeks to generate cartilage organoids (C organoid) followed by 4 weeks of proliferative media (CP organoid) and another 4 weeks of chondrogenic factors (CPC organoids). Control cartilage organoids were kept in chondrogenic media for 8 (CC organoids) and 12 weeks (CCC organoids). (A) schematic of the experiment set‐up. (B) Time course analyses of MSCs makers (CD90, CD44) by FACS of digested cartilage organoids at (a) day 0 and after (b) 4 weeks, (c) 8 weeks and (d) 12 weeks of culture. (N = 8, 4 biological replicates per donor, 2 adult donors tested). FACS data are presented as percentage (mean ± SD) of either the total cell population or the MSCs population. For each time point 4 cartilage organoids per donor (2 adult donor tested) were digested and analyzed by FACS (C) Time course analyses of GAG released during chondrogenic phase 1 (day 0 – C) and chondrogenic phase 2 (CP – CPC). GAG released data is expressed mean ± SD of µg/well (N = 4, 2 biological replicates per donor, 2 adult donors tested). (D) Macroscopic‐, Safranin‐O stained‐ and (E) whole mount‐images (Sox9, Col2, Ki67) obtained after (a) 4 weeks, (b) 8 weeks (CP organoid) and (c) 12 weeks (CPC organoid). (N = 6, 3 biological replicates per donor, 2 adult donors tested). Yellow scale bar = 100 µm)).

**FIGURE 2:**
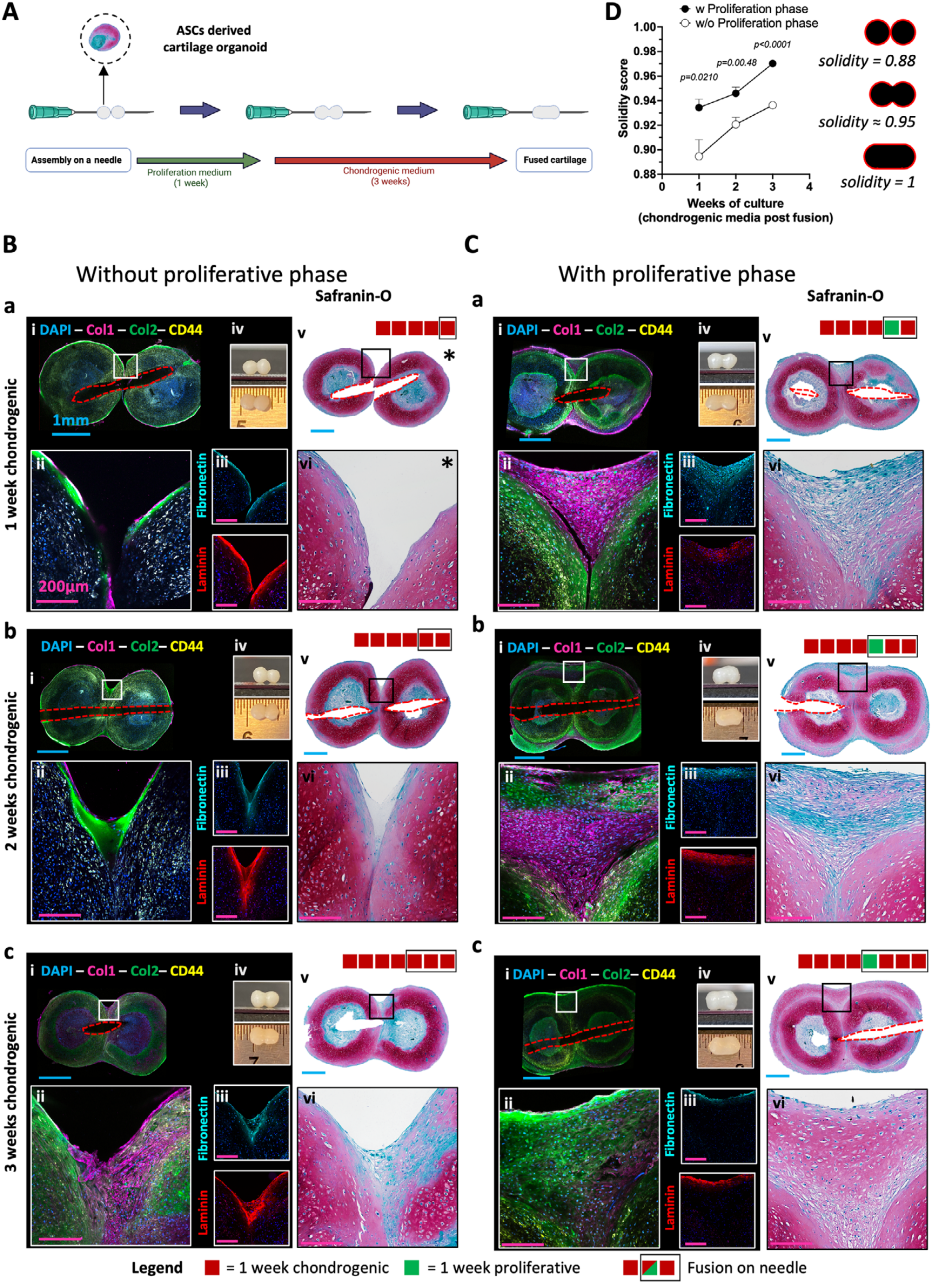
Newly formed perichondrial cell layer enhances cartilage organoids fusion (((A) Schematic of the experimental design. 4 weeks‐ASCs derived cartilage organoids were generated as described in Figure 1. At that time 2 units were assembled together onto a 27G needle. After assembly, cartilage organoids were either exposed (C) or not (B) to proliferative media for 1 week (green square symbol) followed by 3 weeks of chondrogenic factors. Representative images of (i, ii) whole mount staining (Col1, Col2, CD44), (iii) immunofluorescence (Fibronectin, Laminin), (iv) macroscopic and (v‐vi) Safranin‐O staining of fused cartilage organoids after (a) 1 week, (b) 2 weeks or (c) 3 weeks of re‐exposure to chondrogenic factors. (D) Solidity measurements performed on safranin‐O‐stained images. Data are expressed as mean ± SD (N = 3, 3 biological replicates, 1 adult donor tested). For statistical analyses unpaired t test with Welch's correction were used. Blue scale bar = 1 mm and pink scale bar = 200 µm. Red square indicate 1 week exposure to chondrogenic media, green square indicate 1 week exposure to proliferative media, black border indicates fusion and cell culture onto the 27G needle. Star symbol (*) indicates break in fused cartilage organoids resulting from the sample handling. Red dotted lines indicate where the needle was placed.)).

In the context of TE, progenitor cells—used both as a source and resource— are gradually exhausted when generating new tissue in vitro. Here, using an already established protocol to generate ASCs derived cartilage organoids [22], we sought to counteract the exhaustion of progenitor cells. First chondrogenesis is initiated *(Start)* and when cartilage tissue is formed (after 4 weeks) chondrogenic media is removed to interrupt the differentiation process *(Stop)*. At that time, cartilage organoids are exposed to proliferative media (i.e., serum free media (SFM) supplemented with 10% FBS and 5 ng/mL of FGF‐2) to replenish the pool of progenitor cells and re‐establish a layer of undifferentiated cells at the organoid periphery *(Rewind)*. Finally, organoids are re‐exposed to chondrogenic factors to deposit new cartilage matrix at the periphery *(Repeat)*. This matrix is expected to mediate neighboring organoids fusion thus leading to an increase in the overall size of the resulting tissue construct (Figure 1A). FACS characterization showed that the starting ASCs p1 population—obtained from two successive monolayer expansions of stromal vascular fraction (SVF) cells—was homogenous with co‐expression of the stromal mesenchymal surface markers CD90 and CD44 (Figure 1Ba) in line with previous reports [42, 43]. During cartilage organoid formation (4 weeks, C organoids), MSCs gradually turned into chondrocytes and lost the expression of CD90 and CD44 (Figure 1Bb), secreted glycosaminoglycan (GAG) (Figure 1C) leading to the formation of a dense cartilage extracellular matrix containing Sox9 positive and collagen type II (Col2) positive cells characteristic of cartilage tissues (Figure 1Da,1Ea). When stimulating cartilage organoids with proliferative cues (8 weeks, CP organoids), the MSCs fraction is re‐enriched from 26 ± 4 to 52 ± 20% with a higher increase for the double positive (CD90^+^CD44^+^) population from 18 ± 1 to 60 ± 24% (Figure 1Bc). Moreover, when looking at the expression of CD73— a surface marker associated with enhanced chondrogenic and osteogenic potential in MSCs [44, 45, 46]— we could show that a third of CD90^+^CD44^+^‐MSCs population was positive for CD73 (28 ± 7%) and distinct from the starting ASCs p1 population CD90^+^CD44^+^CD73^−^ (92 ± 4%) (Figure S1B). In addition, CP organoids exhibited a thick surrounding layer—termed perichondrial layer—negative for GAG (Figure 1Db) and Col2 with a high cell density containing proliferative chondrogenic progenitors (Sox9^+^Ki67^+^ cells) (Figure 1Eb) which were absent in the control cartilage organoids (8 weeks, CC organoids) (Figure S2A,B). Additional staining by whole mount shows that this perichondrial layer is positive for CD44 and Collagen Type I (Col1) (Figure S3). Interestingly, even after 4 weeks of exposure to proliferative cues, the CP organoids preserved a high GAG content and expression of Col2 within the tissue (Figure 2Db‐Eb) suggesting that the outer layer of the cartilage organoid was the most affected by the exposure to proliferative cues. Upon re‐exposure to chondrogenic factors (CPC organoids), the perichondrial layer was able to give rise to new cartilage as shown by (i) the gradual loss of MSCs expression markers assessed by FACS (Figure 1Bd), (ii) the increase in GAG release (Figure 1C) and (iii) the positive signal for GAG by safranin‐O (saf‐O) and Col2 by whole mount staining (Figure 1Dc‐Ec). In short, by using a dynamic cell culture system that alternates chondrogenic and proliferative cues, it is possible to re‐enrich ASCs derived cartilage organoids with chondrogenic progenitors highlighting the cellular plasticity of these cartilage organoids. This cell population expresses stromal mesenchymal markers, contains Sox9 + proliferating cells and upon re‐exposure to chondrogenic cues generates new cartilage tissue. Whether these progenitors are the results of chondrocytes renewal and/or proliferation of undifferentiated MSCs as yet to be elucidated. However, the expression of CD73 in these MSCs points more toward the emergence of a new mesenchymal population.

### 2.2 Newly Formed Perichondrial Layer Enhances Cartilage Organoid Fusion in Modular Bottom‐up Approach and Enables Scalable and Customizable Cartilage Grafts Production in vitro

In recent years, cartilage organoids are being explored as building blocks in a modular bottom‐up approach to generate larger grafts [32, 33, 34, 35, 36, 47]. In fact, cartilage organoids can easily self‐assemble when two or more organoids are put in contact for a prolonged period of time. However, given the fact that most cartilage organoids are developed in spheroids, achieving a specific size and shape is challenging given the limited surface contact available between two spheres. Here, we sought to utilize the capacity of ASCs derived cartilage organoids to generate a thick perichondrial layer of chondrogenic progenitors to enhance cartilage organoid fusion using a method freely inspired from kenzan bioprinting [48] (Figure 2A). First, cartilage organoids (4 weeks chondrogenic media, 2 units) were assembled together on a 27G needle and exposed (Figure 2C) or not (Figure 2B) to one week of proliferating media followed by 3 weeks of chondrogenic media. The proliferation phase resulted in a thick connective layer of chondrogenic progenitors embedded in a Col1 matrix negative for GAG (Figure S4i–v). Upon re‐exposure to chondrogenic factors, the connective ECM gradually shifted from Col1 to Col2 over the course of 3 weeks resulting in homogenous cartilage tissue (Figure 2C). In contrast, in the absence of proliferating phase, the cartilage fusion was mediated by the migration of few chondrocytes and direct deposition of Col2 ECM in between the two cartilage organoids, resulting in poor tissue integration (Figure 2B). Next, we looked at the expression of fibronectin and laminin—two major ECM proteins supporting cell adhesion, migration, matrix remodeling and chondrogenic differentiation— and found that they were highly expressed on the surface of fusing cartilage organoids (Figures 2B,C iii). Taken together, these results suggest that for cartilage organoids not exposed to a proliferative step, laminin and fibronectin play a role in the organoid fusion where both protein expression colocalized with the one of Col2 (Figure 2B). However, their contribution is likely more limited for cartilage organoids exposed to a proliferation step, where the organoid fusion is mainly driven by the new deposition of Col1 which overtime is remodeled into Col2 (Figure 2C). Using the geometrical solidity— defined as the ratio of the area of an object to the area of a convex hull of the object – on saf‐O‐stained images, we quantified the cartilage fusion efficiency. In between two perfect spheres, the absence of fusion is scored 0.880 in solidity whereas a perfect fusion is scored 1. Our results shows that the proliferative phase enhanced the cartilage organoid fusion with a solidity increase of 0.934 ± 0.007 to 0.970 ± 0.002 versus 0.895 ± 0.014 to 0.936 ± 0.002 in the absence of a proliferative phase (Figure 2D). Interestingly, when compared to the single cartilage organoids cell culture (Figure 1), using the same donors, the cycle of proliferation and re‐exposure to chondrogenic factors was more efficient in the context of cartilage organoid fusion (i.e., larger amount of cartilage obtained) which could be the result of (i) the shorter proliferative phase used (1 week vs 4 weeks), (ii) the local cellular environment of the newly formed connective tissue sandwiched in between cartilage tissues or (iii) a combination of both.

**FIGURE 3:**
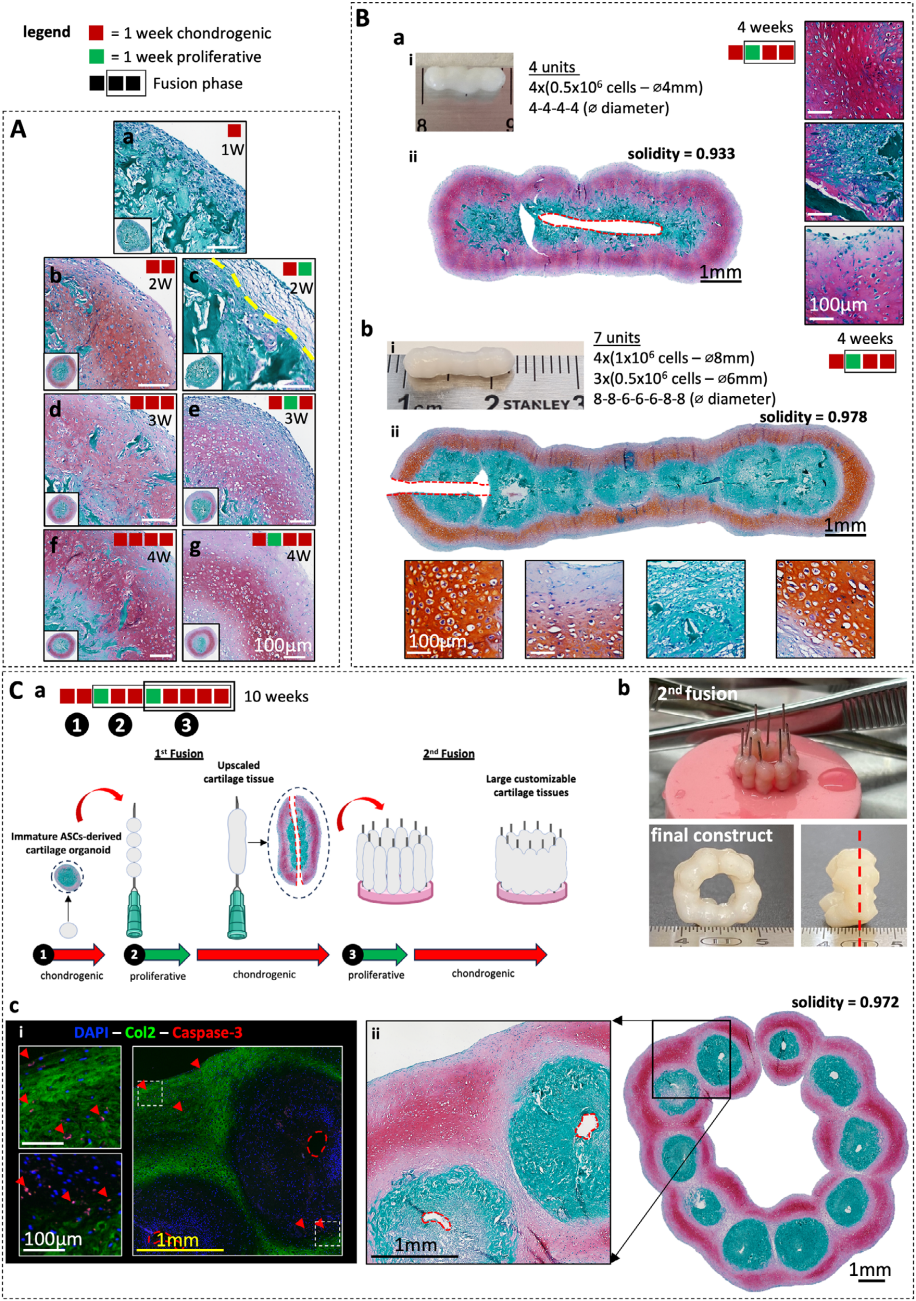
Modular bottom‐up TE approach to generate customizable scaled‐up cartilage grafts in vitro. ((Early proliferative phase during chondrogenesis delays cartilage formation but does not impair chondrogenic capacity. (A) Representative Safranin‐O stained images of cartilage organoids exposed (b,d,f) continuously to chondrogenic media or (c,e,g) with a 1‐week proliferative phase. (N = 6, 2 biological replicates per donor, 3 adult donors tested). (B) Generation of upscaled cartilage grafts (longitudinal axis), immature ASCs‐derived cartilage organoids (1‐week chondrogenic media) are assembled onto a 27G needle and immediately exposed to 1‐week of proliferative media. After proliferative phase, assembled cartilage organoids are re‐exposed to chondrogenic media for 2 weeks to ensure proper cartilage fusion and maturation. Representative (i) macroscopic and (ii) Safranin‐O‐stained images of upscaled cartilage grafts obtained after (a) fusion using 4‐ or (b) 7‐cartilage organoids shaped longitudinally. (C) Iterative approach to generate larger and more complex cartilage grafts in vitro. 10 Fused cartilage grafts (5 weeks)—each obtained from the fusion of 4 immature cartilage organoids (2 weeks chondrogenic induction) —are assembled together to form a ring on a silicon base and exposed to a 1‐week proliferative phase followed by 4 weeks of chondrogenic media (10 weeks total for the duration of the in vitro cell culture). (a) Schematic of the experimental set‐up. Representative (b) macroscopic and (c) (i) Immunofluorescent (DAPI, COL II, Caspase‐3) and (ii) Safranin‐O‐stained images of the final TE cartilage construct. Black scale bar = 1 mm, yellow scale bar = 1 mm and white scale bar = 100 µm. Red square indicates 1 week exposure to chondrogenic media, green square indicates 1 week exposure to proliferative media, black border indicates fusion and cell culture onto the 27G needle. Red dotted lines indicate where the needle was placed.)).

Next, we exploited this enhanced cartilage organoids fusion method to generate larger and more complex grafts in vitro (Figure 3). Following the same principle as in Figures 1 and 2, we showed that early induction of a proliferative phase during chondrogenesis would also enrich the outer layer of the organoids while preserving the capacity to generate cartilage in vitro albeit with a (1 week) delay in production of cartilage matrix (Figure 3A). Then, we generated longitudinal grafts using a 1‐week chondrogenic induction followed by an assembly on the needle, 1‐week proliferative phase and 2 weeks of chondrogenic exposure. We were able to control the length and shape of the constructs by modulating the number and size of cartilage organoids assembled. We found that this protocol was the most robust to generate longitudinal grafts comprised of a thick cartilage outer shell and undifferentiated core (Figure 3B). Not using a proliferative step led to reduced cartilage fusion (Figure S5A,B) while using an extended proliferative step (2 weeks) delayed or prevented cartilage formation (Figure S5C,D). These results were in line with previous reports demonstrating that immature cartilage organoids better self‐assemble [47, 49]. However, for some tested donors we found that a 2‐week chondrogenic induction prior to the fusion was necessary to ensure robust cartilage formation. Finally, we investigated the possibility of iterating the process of proliferative cues followed by chondrogenic induction on already fused cartilage grafts to generate larger and more complex cartilage structures in vitro. To that aim, 10 longitudinal cartilage grafts obtained from 40 immature cartilage organoids were combined to form a macro‐tissue in the shape of a 3D ring demonstrating the tunability of the proposed approach to generate scalable cartilage tissues of complex size and shape (Figure 3C). Interestingly, even in macro‐tissue of this scale, the cellular viability assessed histologically via immunostaining against human cleaved caspase‐3 remained high with only a few positive cells detected per field (Figure 3Cci) and comparable to the one observed in single organoids cell culture (Figure S6Ab‐f) or fused cartilage longitudinal graft (Figure S6B). For all the tested tissues, cell death was detected both within the cartilage (Col2 positive areas) and outside. Finally, compared to other assembloids approaches, the low number of cells, the absence of a hydrogel as a fusing matrix, the customizability and size of the resulting cartilage constructs and their tolerance to hypoxia and limited nutrient availability renders this approach particularly attractive for the TE field.

### 2.3 Phalange Shaped Cartilage Tissues Remodel Efficiently Into Bone Organs via ECO While Preserving their Shape When Implanted in vivo

**FIGURE 4:**
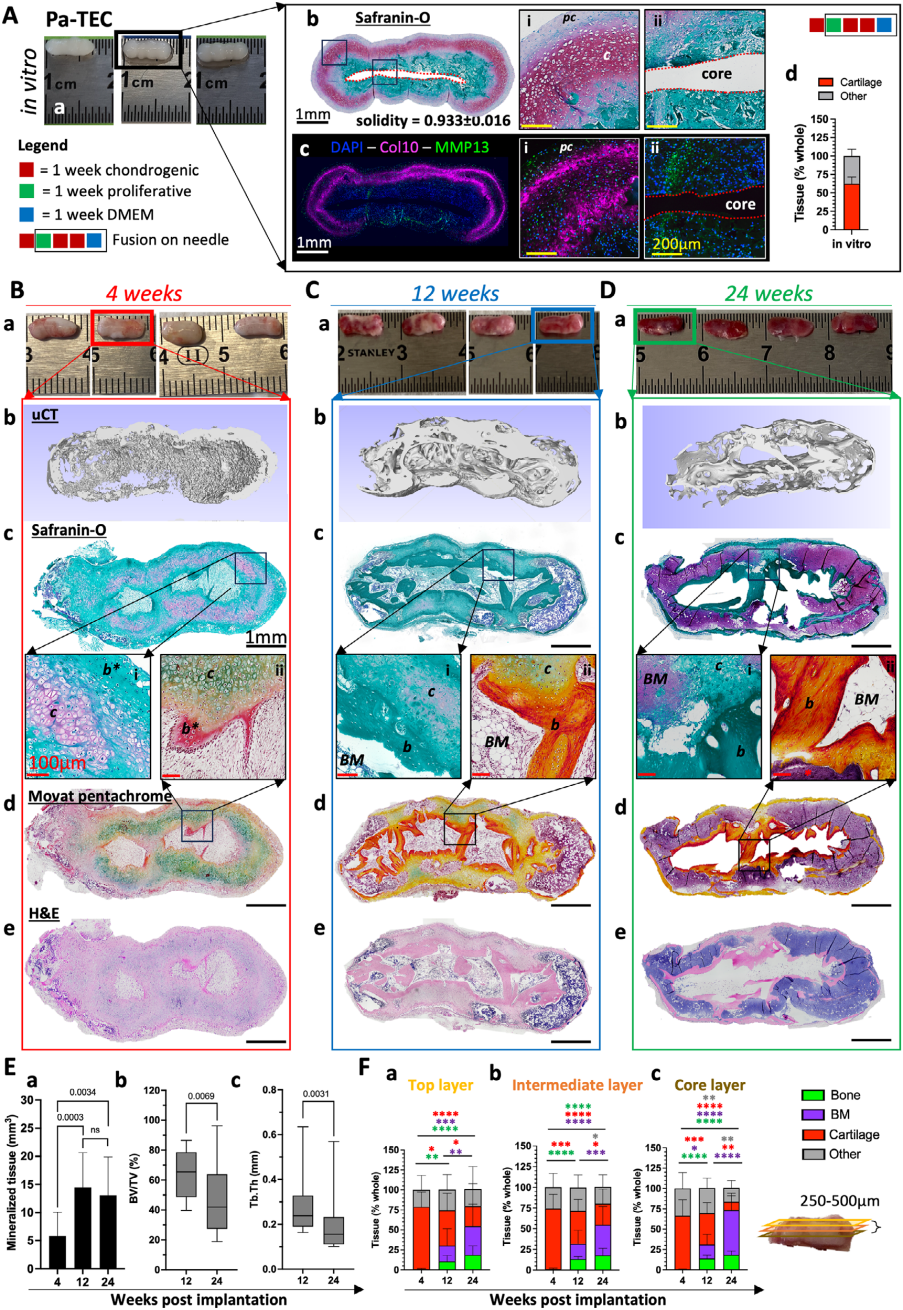
Pa‐TEC robustly recapitulate endochondral ossification forming phalange shaped bone organ when implanted in vivo. ((Pa‐TEC comprised of 4 cartilage organoids are generated and exposed to DMEM media for 1 week before implantation in an ectopic nude mice model for up to 24 weeks. (A) Representative (a) macroscopic, (b) Safranin‐O‐stained and (c) immunofluorescence (Col10, MMP13) images of the Pa‐TEC in vitro. (d) Quantitative assessment of the cartilage area in Pa‐TEC based on safranin‐O staining. Data are expressed as a percentage (mean ± SD) of the whole tissue. (N = 5, 1 biological replicates per donor, 5 adult donors tested). Evolution of the bone remodeling in vivo after (B) 4 weeks, (C) 12 weeks and (D) 24 weeks. Representative (a) macroscopic, (b) µCT 3D reconstruction, (c) Safranin‐O, (d) Movat pentachrome and (e) Hematoxylin and Eosin images after implantation. Evolution of (E) (a) mineralized tissue (mm^3^), (b) Bone Volume / Total Volume (BV/TV in %) and (c) Trabecular thickness (Tb.Th in mm) obtained by µCT. Data are expressed as (mean ± SD). (N ≥ 14, 2–4 biological replicates per adult donor per time point, 5 adult donor tested). For statistical analyses one‐way ANOVA with Tukey's multiple comparisons tests was used (ns, p>0.05) for the mineralized tissue (mm^3^) quantification and Mann Whitney test on BV/TV and Tb.Th measurements (ns, p>0.05). Evolution of (F) Bone, Bone Marrow, Cartilage and Other tissues quantification within the H&E sections at 3 different depths: (a) Top Layer, (b) Intermediate layer and (c) Core layer after 4, 12 and 24 weeks in vivo. On panel (F) the statistical significances are represented with stars rather than the p values for clarity. (*p < 0.05), (**p < 0.01), (***p < 0.001), (****p < 0.0001). Legend: red square indicates 1 week exposure to chondrogenic media, green square indicates 1 week exposure to proliferative media, black border indicates fusion and cell culture onto the 27G needle, blue square indicates 1 week exposure to DMEM media. Red dotted lines indicate where the needle was placed. Symbols, cartilage (c), perichondrium (pc), bone (b), immature bone (b*) and bone marrow (BM). Black scale bar = 1 mm, white scale bar = 1 mm, yellow scale bar = 200 µm andred scale bar = 100 µm.)).

Next, we investigated the possibility to use our modular approach to generate grafts suitable for the treatment of children suffering from symbrachydactyly—a rare, congenital limb abnormality that results in short or missing fingers [50, 51, 52, 53]. Longitudinal cartilage grafts comprised of 4 units imitating the shape and size of small phalanges were generated following the same method described in Figure 3B. We termed these grafts Pa‐TEC for Phalange shaped adipose‐derived Tissue Engineered Cartilage. To assess the Pa‐TEC bone forming capacity, we used an ectopic immuno‐compromised mice model mimicking the clinical scenario of an empty phalangeal pocket and the autologous envisioned TE strategy (Figure 4). One week prior to the implantation, the chondrogenic media was removed and replaced by DMEM to prime the cartilage toward hypertrophy. At that time, Pa‐TEC were comprised of large hypertrophic cartilage shell expressing collagen type X (Col10) and matrix metalloproteinase‐13 (MMP13), a soft core of undifferentiated progenitor cells and a perichondrium peripheral layer (Figure 4A). In vivo, the ECO remodeling process was robust and reproducible for all the tested donors with formation of bone organs analog to the one occurring during the development of long bones where cartilage begets bone and endochondral myelopoiesis [54]. Specifically, we tested 5 adult donors (30–56 years old, 1 male donor, 4 female donors) and 3 pediatric donors (12 ‐25 months old donors, all male) to assess the robustness of the Pa‐TEC bone remodeling. For each donor, 12–16 Pa‐TEC were generated and either kept in vitro (*n = 2‐3*) or implanted in vivo and explanted after 4 (4w), 12 (12w) and 24 (24w) weeks (*n = 2‐4*; per time‐point). Figure 4B summarizes the in vivo data obtained for adult donors, whereas the individual ECO potential for each donor is presented in Figure S7 for the adult and in Figure S8 for the pediatric donors. Figure 4E summarizes the data obtained by micro‐computed tomography (µCT) including the amount of mineralized tissue obtained, the ratio bone volume / total volume (BV/TV) and the trabecular thickness (Tb.Th) measurements. However, such µCT measurements were inefficient to properly segment bone tissue from mineralized cartilage in vivo. Therefore, in an effort to better characterize the ECO remodeling and accurately quantify the bone formation, tissue sections from 3 different depths (each spaced 250–500 µm apart) corresponding to the top‐, intermediate‐ and core‐layer were generated and analyzed histologically (Figure 4F). During the first weeks of implantation, implanted tissues began mineralizing from the perichondrial layer, concomitantly immature trabecular bone formed first in apposition to the cartilage at the core of the implanted Pa‐TEC (Figure 4B). Later, between 4 and 12 weeks, most of the bone network was formed as attested by the increase in mineralization from 5.83 ± 4.23% to 14.45 ± 6.20% (Figures 4C–E), the high BV/TV observed at 12 weeks (64.10 ± 15.81%) and the amounts of bone detected on HE sections were 0.47 ± 0.74% to 12.44 ± 4.79% for the 4w‐ and 12w‐timepoint, respectively (Figure 4Fc). While the bone network was formed, cartilage tissue was gradually replaced by bone marrow cavities with a decrease of cartilage content from 66.13 ± 19.56% to 39.06 ± 11.57% and then 16.71 ± 20.49% mirrored by the apparition of bone marrow tissues from 0.07 ± 0.26% to 16.13 ± 11.51% and then 53.14 ± 25.74% for the 4w‐, 12w‐ and 24w‐timepoint, respectively (Figure 4Fc). After 24 weeks, the implanted Pa‐TEC had remodeled into bone organs with a cortical bone shell, surrounding large bone marrow cavities and a dense and mature inner trabecular network with little to no remanence of cartilage tissues (Figures 4D–F; Figure S7D). At that time, while the amount of mineralized tissue was not as significantly reduced as compared to the 12w‐timepoint, the reduction in BV/TV (down to 45.42 ± 22.25%) reflected the disappearance of mineralized cartilage observed histologically while the reduction in Tb.Th. (from 0.28 ± 0.13 to 0.18 ± 0.12) likely indicated early stages of bone remodeling (Figure 4E).

For pediatric donors, despite a lower chondrogenic potential characterized by a greater heterogeneity and a reduced cartilage formation (37.92 ± 9.45% of the tissue, vs. 62.48 ± 9.15% for the adult donors) (Figure S8F), pediatric Pa‐TEC were also able to remodel into bone organs following the same ECO process. Notably, the remodeling speed was slower for pediatric Pa‐TEC with a relative high persistence of cartilage tissue (30.81 ± 22.25%) concomitant with reduced bone marrow content (20.86 ± 11.58%) and robust bone formation (18.45 ± 10.95%) for the core layer after 24 weeks of implantation (Figure S8D,Gc). Compared to our previous research, the size and quality of hypertrophic cartilage grafts generated from ASCs and their bone remodeling are significantly improved [22, 41]. Moreover, the specific size, cartilage patterning of the Pa‐TEC—hypertrophic cartilage shell surrounding undifferentiated progenitor cells embedded in the collagen sponge scaffold—and the slow in vivo remodeling contributed to a more faithful recapitulation of ECO highlighting its potential both as a clinical approach and as a model to study ECO.

### 2.4 Contribution of Implanted and Host Recruited Cells to Bone Formation and Remodeling

**FIGURE 5:**
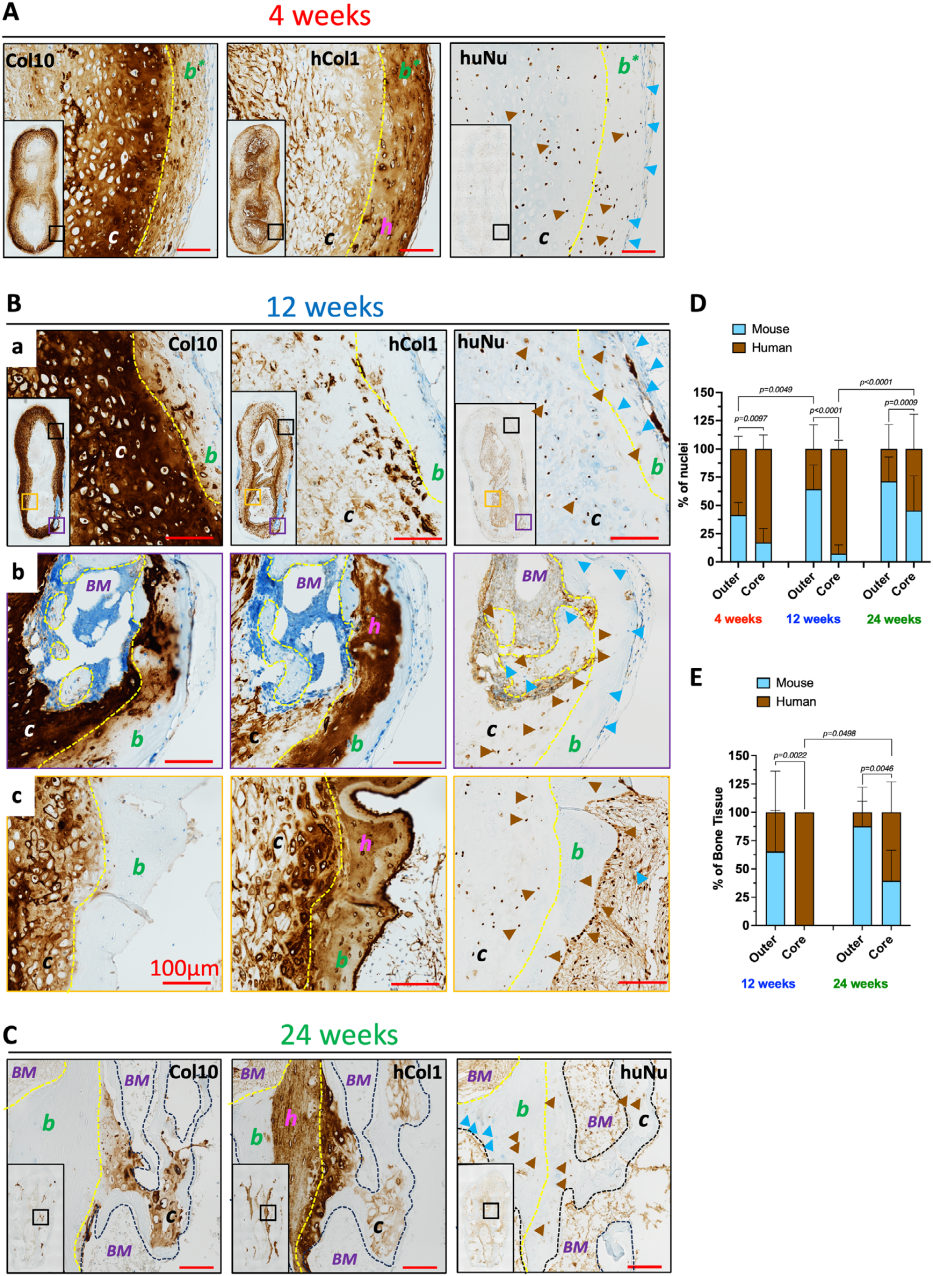
Implanted human‐ and host recruited murine‐cells contribution to endochondral ossification and bone remodeling. ((Human implanted cells initiate bone formation both on the outer and core parts of the Pa‐TEC while mouse recruited cells create bone marrow compartments and mediate bone remodeling over time. Immuno‐histochemistry staining of collagen type X (Col10), human collagen type I (hCol1) and human Nuclei (huNu) in Pa‐TEC (adult donors) after (A) 4 weeks, (B) 12 weeks and (C) 24 weeks of implantation. (D) Human and mouse nuclei quantification in outer and core areas of the implanted Pa‐TECs performed on huNu stained images. Data are expressed in percentage of nuclei (mean ± SD). (N ≥ 15 images per time point per area, 1–2 biological replicate per donor, 3 adult donors tested). (E) Evolution and quantification of hCol1 in bone tissues of the implanted Pa‐TECs after 12 and 24 weeks performed on hCol I stained images. Data are expressed in percentage of bone tissue (mean ± SD). (N ≥ 5 Pa‐TEC per time point, 1–2 biological replicate per donor, 5 adult donors tested). For statistical analyses one‐way ANOVA with Tukey's multiple comparisons tests were used. Blue arrow indicates mouse cells (blue nuclei), black arrows indicates human cells (brown nuclei). Red scale bar = 100 µm. Symbols, cartilage (c), bone (b), immature bone (b*), bone marrow (BM) and human (h). Yellow dotted lines indicate bone or immature bone areas.)).

A critical factor in bone TE is ensuring that the engineered graft integrates with, and is eventually replaced by, the host own tissue [55]. Therefore in this study, we investigated the contribution of human implanted cells and host‐recruited murine cells to the bone formation and remodeling during the ECO process. Specifically, we stained for human nuclei (huNu) to detect the presence of the implanted cells, human collagen type I (hCol1)—the most abundant collagen in bone tissue— to detect the newly formed bone tissue of human origin and collagen type X (Col10) to detect hypertrophic cartilage in representative samples of the adult Pa‐TEC in vivo data set (Figure 5; Figure S9). Here, our data revealed that the human implanted cells are responsible for initiating the bone formation (highlighted by the deposition of hCol1) at periphery and the core of the implanted Pa‐TEC (Figures 5A–C). Next, the host recruited cells invaded the implanted constructs and remodeled the human bone matrix starting from the periphery (between week 4 and week 12), and later at the core (between week 12 and week 24) (Figures 5B–D). Quantitative analysis of the huNu and hCol1 confirmed this human to mouse bone remodeling with increased proportion of mouse cells and mouse derived bone throughout the implantation period, demonstrating the successful integration and, eventually, replacement of the Pa‐TEC by host bone tissue (Figures 5D,E). In previous reports, we described the outer/cortical bone to be of mouse origin because of the presence of mouse cells embedded in the bony matrix. However, these studies did not assess the ECO at an early in vivo time‐point (4 weeks in this study) [41, 56]. Further experiments would be needed to determine the origin of the human bone forming cells whether they arise from hypertrophic chondrocytes trans‐differentiation or progenitor cells still present in the Pa‐TEC at the time of implantation. Nevertheless, these data highlight the synergic contribution of implanted and host‐recruited cells to generate a functional bone organ.

### 2.5 Assessing the Predictability and Biomechanical Performance of Implanted Pa‐TEC for Future Clinical Use

**FIGURE 6:**
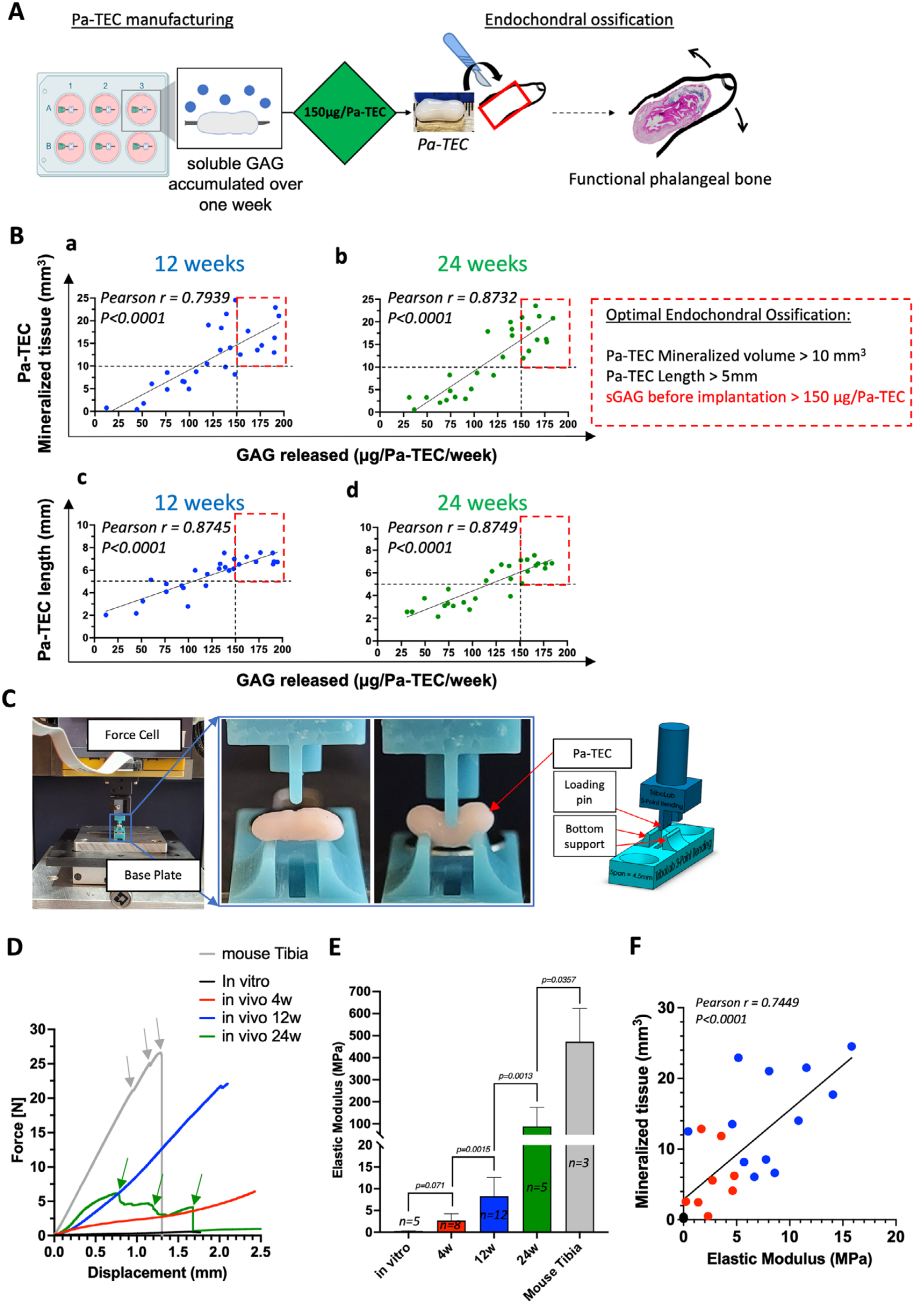
Pa‐TEC release criterion and biomechanical properties ((A) Schematic of Pa‐TEC production and clinical application. (B) GAG release in spent DMEM media accumulated for one week and sampled on the day of implantation as a predictive marker for ECO potential. Pearson correlation between GAG release (µg/Pa‐TEC) and (a,b) the mineralization (mm^3^) and (c‐d) the explanted Pa‐TEC length assessed by µCT after (a,c) 12 weeks and (b,d) 24 weeks of implantation. (N = 27, 2–4 biological replicates per donor per time point, 8 donors tested (5 adult donors and 3 pediatric donors)). Pa‐TEC are mechanically apt during bone remodeling (C) Schematic of the 3‐point bending test experimental set‐up. Evolution of (D) the displacement curves and (E) the Elastic modulus of Pa‐TEC in vitro and in vivo. Mouse tibia was used as a reference. Data are expressed in MPa (mean ± SD). (N ≥ 5, 1–3 biological replicates per adult donor per time point, 3 adult donor tested). For statistical analyses one‐way ANOVA with Tukey's multiple comparisons tests were used. (F) Pearson correlation between the elastic modulus (MPa) and the mineralization (mm^3^) assessed by µCT before implantation (black dots) and after 4 weeks (red dots) and 12 weeks (blue dots) of implantations (N = 25).

Given the nature of the envisioned clinical application—phalange construction in children suffering from symbrachydactyly—we investigated (i) GAG released (µg/Pa‐TEC/week) as a potential release criterion for efficacy (Figure 6B) and (ii) whether Pa‐TEC could be mechanically apt in supporting digit movement (Figures 6C–F). Interestingly, the ECO capacity of each individual Pa‐TEC, from both adult and pediatric donors, could have been predicted in vitro by measuring the amount of GAG accumulated in vitro for 1 week in the DMEM phase before implantation which strongly correlated (r = 0.8732, *p* < 0.0001 after 24 weeks) with the mineralized content (Figure 6Ba,b) and with the explanted Pa‐TEC length (r = 0.8749, *p* < 0.0001 after 24 weeks, Figure 6Bc,d) measured by µCT at both 12w‐ and 24w‐in vivo time‐points assessed. Poorly differentiated Pa‐TEC characterized by a GAG released inferior to 100 µg/Pa‐TEC were prone to resorption. In contrast a Pa‐TEC with a GAG released superior to 150 µg/Pa‐TEC exhibited robust bone formation (mineralized volume > 10mm^3^) accompanied with maintenance of shape and size (length > 5 mm). Moreover, given the non‐destructive nature of the assay, it could easily be implemented in the future as a potential release criterion to guarantee the success of the Pa‐TEC bone remodeling in a clinical setting.

Next, to assess their biomechanical properties, in vitro and in vivo adult Pa‐TEC were evaluated in a custom‐made 3‐point bending test (Figure 6C). The biomechanical properties of the Pa‐TEC were reflective of their tissue composition described in Figure 4. In vitro Pa‐TEC were very elastic, bending easily without breaking under the pressure of the loading pin with a maximal stress of 0.56 N (Figure 6D). After 4 and 12 weeks of implantation, despite high mineralization content, we observed a similar profile (i.e., smooth deformation of the grafts without breaks) albeit with higher recorded maximum stress 6.42 and 22.1N for the 4w; and 12w time‐points due to the persistence of cartilage tissue (Figure 6D). In contrast, after 24 weeks in vivo Pa‐TEC—now mature bone organs where the cartilage has been remodeled into bone marrow compartments—were prone to break during biomechanical testing as indicated by the successive drop in the displacement curve and recorded a maximal stress of 6.20 N (Figure 6D). To quantify the increase in stiffness during the ECO processes we calculated the elastic modulus for each tested Pa‐TEC based on the slope of the elastic region of the recorded strain. We could show that the stiffness of the Pa‐TEC was steadily increasing during the ECO process from 0.26 ± 0.10 MPa before implantation to 87.75 ± 88.59 MPa after 24 weeks in vivo. While it represents a dramatic increase of the stiffness, it remains lower than 472.11 ± 150.74 MPa obtained when testing mouse tibias (Figure 6E). This discrepancy between the TE bone and the native mouse bone can be partially explained by the absence of mechanical stimulation during and after the ECO remodeling of the grafts in the in vivo ectopic model. In addition, we could show that the Elastic modulus correlated *(r = 0.7449*, *p* < 0.0001) with the mineralization content measured by µCT (Figure 6F). Finally, both the identified release criterion (GAG released prior to implantation) and the biomechanical properties of the Pa‐TEC demonstrate the relevance of the proposed strategy for the envisioned clinical translation.

## 3 Conclusion

In the herein study, we report a scalable, tunable bone TE strategy for the treatment of congenital and large bone defects based on the self‐assembly of adipose‐derived cartilage organoids. We demonstrate how chondrogenic progenitors can be replenished after chondrogenic differentiation and subsequently re‐differentiated thereafter in a ‘*Start, Stop, Rewind, Repeat’* approach. Next, we utilize this cellular plasticity to generate perchondrial layers that act as a biological cement to efficiently assemble several cartilage organoids into larger and more complex grafts. Moreover, we show how each resulting scaled up cartilage graft can itself be used as a biological building block to further increase size and/or complexity by simply iterating the proliferative and chondrogenic phases emphasizing the *Repeat* function of the proposed approach.

This TE strategy presents several advantages for a potential clinical translation and first in‐human application. (i) ASCs are an easily available cell source suitable for both adult and pediatric settings. (ii) The in vitro manufacturing is straightforward, customizable in size and shape—potentially meeting patient‐specific needs—, robust and easily adaptable to a GMP setting. (iii) ASCs‐derived cartilage organoids used as building blocks are sizable (5‐15 mm^3^) and require a low number of progenitor cells compared to microaggregates strategies [33, 36, 47]. (iv) Assembly/fusion steps are mediated by new ECM deposition and thus do not require additional filling material such as hydrogel resulting in a mechanically apt—capable of withstanding compression forces without breaking—homogenous cartilage tissue. (v) In vivo, these grafts (termed Pa‐TEC) recapitulate the developmental ECO where cartilage begets bone and endochondral myelopoiesis through a synergic collaboration between implanted and host‐recruited cells to generate over time a fully integrated bone organ analog to the native bone in tissue architecture and mechanical strength. (vi) Last but not least, we demonstrate that the soluble GAG content in spent media can be used as a reliable biological marker to predict the bone forming capacities of such grafts after implantation.

For these reasons, we believe that the proposed TE strategy could be used in the near future to engineer autologous Pa‐TEC to treat children suffering from symbrachydactyly as a first in‐human study.

## 4 Limitations of the Study

The ectopic non‐loaded in vivo model used in this study accurately mimics the envisioned clinical scenario of an empty phalangeal pocket, in which surrounding bone and mechanical loading would be mostly absent. However, to broaden the translational potential of this TE strategy, further in vivo studies are required in clinically‐relevant orthotopic and mechanically‐loaded settings to assess host‐construct interface integration, bone and enthesis formation and joint surface load adaptation. Herein, the relatively small size of the Pa‐TEC—though suitable for the treatment of children suffering from symbrachydactyly— did not require pre‐vascularization in order to achieve robust ECO. In larger TE cartilage grafts, it remains to be proven if pre‐vascularization is required for efficient bone remodeling. Finally, another limitation concerns the origin of the arising MSCs population in the ASC‐derived cartilage organoids in response to proliferative cues which is not fully elucidated in the present study. In order to clearly establish the origin and heterogeneity of such populations, lineage tracing and single cell sequencing experiments should be conducted. Nevertheless, regardless of their origin, their beneficial role on cartilage organoids fusion through the formation of a chondrogenic perichondrial layer is clearly demonstrated.

## 5 Experimental section

### 5.1 Cell Isolation and Cell Culture

Adipose tissues and lipoaspirates were obtained under the general hospital consent (University Hospital of Basel, Switzerland), following informed consent from the patient, and in accordance with the Ethical Committee of Northwest and Central Switzerland (EKNZ) following the Article 2(2)(c) of the Swiss Human Research Act (HRA, RS 810.30). Adult donors (7 donors, 1 male, 6 female age range 30–74 years old) and pediatric donors (3 donors, all male, age range 13.5‐29‐month‐old) were used in the study. For all tested donors, SVF cells isolated from either adipose tissue or lipoaspirate were recovered following a protocol previously described [57]. After isolation, SVF cells were seeded at a density of 10 000 cells/cm^2^ in complete medium (CM), consisting of α‐MEM (Gibco, 22571‐038) supplemented with 10% fetal bovine serum (Gibco, A5256701), 1% HEPES (Gibco, 15630‐056), 1% sodium pyruvate (Gibco, 11360‐039), 1% penicillin‐streptomycin‐glutamine (Gibco, 10378‐016), as well as 5 ng/ml FGF‐2 (Bio‐Techne, 233‐FB‐025) and further cultured at 37°C, 5% CO_2_ and 95% humidity with media change twice a week. Upon reaching confluency (90%), cells were detached with 0.05% trypsin/0.01% EDTA (Gibco, 25300‐054) and seeded again at 3 000 cells/cm^2^ for one additional cell passage. The resulting population was termed ASC p1.

### 5.2 Collagen Scaffold Preparation

Cylindrical collagen sponge scaffolds were obtained from the commercially available Avitene Ultrafoam Collagen Sponge from Becton Dickinson (Ref#1050050, lot number #WBDW001 and #WBHW0031). Briefly, biopsy punch of either ⌀ 4‐, 6‐ or 8‐mm were made out of the collagen Ultrafoam sheet (8cmx12.5cm h 3mm) and kept dry and sterile at 4°C until used for cell culture.

### 5.3 Cartilage Organoids Formation

Unless stated otherwise, cartilage organoids were generated as follow: ASC p1 (0.5 × 10^6^ cells) seeded onto cylindrical ⌀ 4 mm x h 3 mm collagen sponge scaffold placed in 12‐well plates (TPP, 92412) coated with agarose 2% (Sigma, A‐9539) and exposed to chondrogenic media. Chondrogenic media was composed of serum‐free culture medium (SFM) supplemented with 10^−7^ M dexamethasone (Sigma, D‐2915), 0.1 mm ascorbic acid (Sigma, A‐8960), 10 ng/ml transforming growth factor‐β3 (TGF‐β3, PeproTech, 100–36E), and 10 ng/ml bone morphogenetic protein 6 (BMP‐6, PeproTech, 120‐06). SFM medium was composed of Gibco Dulbecco's Modified Eagle Medium (DMEM, Gibco, 10938‐025), 1% HEPES (Gibco, 15630‐056), 1% sodium pyruvate (Gibco, 11360‐039), 1% penicillin‐streptomycin‐glutamine (Gibco, 10378‐016), 1% ITS+1 (Sigma, I2521), and 1.25 mg/ml human serum albumin (CSL Behring, 43075). Immature cartilage organoids were kept for 1 to 2 weeks in culture whereas mature cartilage organoids were kept in culture for 4 weeks at 37°C, 5% CO_2_ and 95% humidity with media change twice a week. When applicable, chondrogenic media was replaced by SFM proliferative media (i.e., SFM medium supplemented with 10% FBS and 5 ng/ml FGF‐2 to stimulate cartilage resident cells proliferation for 1 to 4 weeks depending on the experiments. Larger cartilage organoids were generated following the ⌀ 4 mm collagen sponge protocol, with the following cell seeding density (i) 0.5 × 10^6^ cells for ⌀ 6 mm x h 3 mm collagen sponge and (ii) 1 × 10^6^ cells for ⌀ 8 mm x h 3 mm collagen sponge (Figure 3Bb).

### 5.4 Cartilage Organoids Fusion and Pa‐TEC Generation

Immature or mature cartilage organoids (min 2 to max 7 units) were pierced through the center and assembled onto a 27G needle. After assembly, cartilage organoids were exposed to SFM proliferative media for 1 week before being re‐exposed to chondrogenic media for 2 weeks unless stated otherwise. Media volume was adjusted for each organoid fusion depending on the number of cartilage organoids assembled (2 mL per cartilage organoid assembled). For the generation of the cartilage ring (Figure 3C), 20 × 10^6^ ASCs p1 were used to generate 40 immature cartilage organoids (2 weeks). Next, 10 longitudinal grafts (4 units each) were assembled and exposed to one‐week SFM proliferative media followed by 2 weeks chondrogenic media (first fusion). At that time, the 10 upscaled grafts were positioned and secured in a circle on a silicon disk transferred to a large cylindrical container (200 mL capacity, 50 mL of media used) and exposed to SFM proliferative media followed by 4 weeks of chondrogenic media (second fusion). Phalange‐shaped adipose‐derived Tissue Engineered Cartilage grafts (Pa‐TEC) were generated from 4 immature cartilage organoids (1 to 2 weeks chondrogenic exposure before assembly depending on the chondrogenic capacity of the donor), assembled onto a 27G needle and exposed to SFM proliferative media for 1 week followed by 2–5 week of chondrogenic media to ensure proper cartilage maturation (8 mL/Pa‐TEC). For the Pa‐TEC, after cartilage maturation, chondrogenic media was removed and replaced by DMEM (Gibco, 11995‐065) devoid of growth factors for one week without media renewal to prepare the grafts for implantation. Regardless of the media composition, cell cultures were kept at 37°C, 5% CO_2_ and 95% humidity with media change twice a week unless otherwise stated.

### 5.5 Animal Experiments

After the one‐week DMEM phase, Pa‐TEC (maximum 4 per mouse) were implanted subcutaneously in athymic CD1 nu/nu female nude mice (Janvier, Rj:NMRI‐*Foxn1^nu/nu^*). Mice were operated under the permission of the Cantonal Veterinary Office of Basel‐Stadt (permit number BS 1797, and National no. 37404) as previously described [18]. At the prescribed time‐points 4‐, 12‐ and 24‐weeks post‐implantation, mice were euthanized and implanted Pa‐TEC were recovered and fixed in Paraformaldehyde 4% (Thermo Scientific, 28906) overnight at 4°C.

### 5.6 Flow Cytometry Analyses

To identify mesenchymal stromal cells populations within ASCs‐derived cartilage organoids, we performed flow cytometry analyses at d0, 4‐ 8‐ and 12‐weeks of culture. Briefly, at the prescribed timepoints, cartilage organoids were minced in small pieces using a scalpel. Then, tissue fragments were digested in PBS with 1.5% Type II Collagenase (Worthington Biochemical, Cat#LS004176) on an orbital shaker (300 rpm, 37°C, 40 min). The resulting cell suspension was washed and resuspended in a flow cytometry buffer containing PBS with 2% FBS and 2.5 mM EDTA. Cells were incubated with the following antibodies for 20 min at 4°C: human anti‐CD90 (Abcam, Cat#ab272351), human anti‐CD44 (BD Pharmingen, Cat#559942) and human anti‐CD73 (BD Pharmingen Cat#550257), all at 1:200 dilution. After staining, cells were washed once with flow cytometry buffer and fixed for 20 min using the Cytofix/Cytoperm Fixation/Permeabilization Kit (BD Bioscience, Cat#554714). After fixation, stained cells were rinsed once more with flow cytometry buffer and kept at 4°C until analysis. The following day, cells were filtered through a 40 µm strainer before acquisition. Samples acquisition was performed on CytoFLEX (Beckman Coulter Life Sciences, Indianapolis, USA) and data were analyzed using FlowJo software (v.10).

### 5.7 Whole Mount Stainings

ASC‐derived cartilage organoids were fixed in Paraformaldehyde 4% (Thermo Scientific, 28906) overnight at 4°C. After fixation, samples were washed once and kept in PBS until staining. Before staining, samples were cut in half using a scalpel and permeabilized in PBS + 0,4% Triton X‐100 solution (Sigma‐Aldrich, Cat#93443) for 10 min at room temperature (RT), followed by incubation in a blocking solution (PBS + 0,1% Triton X‐100 + 5% goat serum) for 1 h at RT. Next, samples were incubated overnight at 4°C with the following primary antibodies diluted in PBS + 0,1% Triton X‐100 : anti‐human Collagen Type I (Abcam, Cat#ab138492), anti‐human Collagen Type II (Invitrogen, Cat#MA5‐12789), anti‐human CD44 (BD Pharmingen Cat#559942), anti‐human Ki67 (Invitrogen, Cat#14‐5698‐82) and anti‐human Sox9 (Millipore, Cat#AB5535). After being washed three times for 15 min with PBS + 0,1% Triton X‐100, samples were incubated for 1 h at RT with secondary antibodies diluted 1:300 in PBS + 0,1% Triton X‐100 : Alexa Fluor 488 goat anti‐rabbit IgG (H+L) (Invitrogen, Cat#A‐11008), Alexa Fluor 546 goat anti‐mouse IgG2a (Invitrogen, Cat#A‐21133), Alexa Fluor 488 goat anti‐rat IgG (H+L)(Invitrogen Cat# A‐11006), Alexa Fluor 546 goat anti‐rabbit IgG (H+L) (Invitrogen, Cat#A‐11010) and Alexa Fluor 647 goat anti‐mouse IgG (H+L)(Invitrogen A‐21235). Nuclei were stained with a DAPI solution (1:1000) in PBS for 30 min and washed once with PBS. Samples were stored in PBS at 4°C until imaging. Microscopic imaging was performed using the Nikon AX/AXR confocal microscope.

### 5.8 sGAG Release in Supernatants

Supernatants from cartilage organoids were collected every week during chondrogenic differentiation phases to quantify released soluble sGAG. At that time, sGAG were accumulating for 3 days before sampling. In addition, Pa‐TEC supernatants from the DMEM phase (i.e., last week of cell culture before implantation) were collected. For Pa‐TEC, sGAG were left accumulating for one week before sampling occurred on the day of implantation. Supernatants were kept at ‐20°C until analysis. Quantification of sGAG was performed using the Blyscan sulfated Glycosaminoglycan assay kit (Biocolor, Cat#B1000) following the manufacturer's protocol. Absorbance readings were performed at 656 nm using the H1M Biotek Synergy plate reader (Agilent, Santa Clara, USA).

### 5.9 Microcomputed Tomography

For microcomputed tomography (microCT), fixed samples were acquired using the high‐resolution scanner (SkyScan1172, Skyscan, Belgium) and 0.5 mm aluminum filtered X‐rays (applied voltage 50 kV; current, 200 µA). Transmission images were acquired during a 360° scan rotation with an incremental rotation step size of 0.25°. Reconstruction was performed using a modified Feldkamp algorithm at an isotropic voxel size of 5 µm. 3D rendering, thresholding, segmentation and 3D measurements were performed using VG Studio MAX 2.2 software (Volume Graphics, Heidelberg, Germany). In particular, bone volume over total volume (BV/TV) and the trabecular thickness (Tb.Th) measurements were obtained using the software's surface determination functionality with a 10000 gray values threshold to segment bone and background within the Pa‐TEC defined as region of interest (ROI).

### 5.10 Histological Stainings and Image Acquisition

For histological analyses, fixed samples were decalcified by 15% EDTA solution if necessary, and embedded in paraffin. Samples were cut into 5 µm thick sections using a Microtome HM 355S (Thermo Scientific) and the sections were placed onto either Polysine adhesion Microscope slides (Epredia, P4981001) or Histobond+M slides (Biosystems, 0811701) for immuno‐histochemistry and immunofluorescence. The tissue sections were deparaffinized and rehydrated then stained with Haematoxylin‐Eosin (both from Sigma Aldrich) and Safranin‐O (Sigma‐Aldrich, 84120) using the Epredia Gemini AS Automated Slide Stainer. Movat pentachrome staining were performed using the kit (StatLab, KTRMP) and following manufacturer's instructions.

#### 5.10.1 Immunofluorescence Analyses

For immunofluorescence analyses, tissue sections were deparaffinized, rehydrated and processed with heat‐induced antigen retrieval using Q Retrieval Low pH 6.0 Citrate Buffer (Quartett, #AR‐001‐0120) for 20 min at 95°C. Tissue sections were permeabilized with 0.5% Triton X‐100 in PBS for 10 min at room temperature (RT) and saturated with blocking buffer (0.1% Triton X‐100 in PBS + 5% Goat Serum and 2% Bovine Serum Albumin) for 1 h at RT. Primary antibodies incubation was performed in a humidified, dark chamber overnight at 4°C. The following primary antibodies were diluted at 1:200 (unless otherwise specified) in blocking buffer: anti‐human Collagen Type X (Invitrogen, #14‐9771‐82, dilution 1:100), anti‐human MMP13 (Abcam, #ab39012), anti‐human Cleaved Caspase‐3 (Abcam, #ab39012), anti‐human Collagen Type II (Merck Millipore, #MAB8887), anti‐human Laminin (Abcam, #ab11575) and anti‐human Fibronectin (Abcam, #ab2413). The next day, corresponding secondary antibodies were added at a dilution of 1:200 in blocking buffer for 1 h at RT: Alexa fluor 647 goat anti‐mouse IgG1 (Invitrogen, #A21240), Alexa fluor 546 goat anti‐rabbit IgG (H+L) (Invitrogen, #A11035). Nuclei staining was done using DAPI solution (BD Pharmingen, #564907) diluted at 1:1000 in blocking buffer for 5 min at RT. Slides were mounted with a coverslip using Fluoromount Aqueous Mounting Medium (Sigma‐Aldrich, #F4680) before imaging. As a positive control for the Caspase‐3 staining, we used cartilage organoids treated with 0.1 µM of Staurosporine (Merck Millipore 569397) for 24 h to induce cell death. Immunofluorescent images were acquired with a Nikon Ti2 widefield microscope, a Photometrics 95B (25 mm, back‐illuminated sCMOS) camera and a CFI Plan Apo Lambda NA 0.45, 10X objective. The software used was the NIS‐Elements AR 6.20.02.

#### 5.10.2 Immunohistochemistry Staining

Human Collagen Type I (hCol1) (Reactivity: human, Abcam, ab138492), Collagen Type X (Col10) (Reactivity: human, Invitrogen, 14‐9771‐80) and human Nuclei (huNu) (Reactivity: human, Merck MAB4383) stainings were performed with Ventana Discovery Ultra (RocheDiagnostics, Switzerland, SA) automated slide stainer. In brief, tissue sections were deparaffinized and rehydrated. Antigens were retrieved by a protease (Protease 3, ref. 760–2020, Ventana) digestion for 20–44 min at 37°C. Primary antibody was manually applied and incubated for 1 h at 37°C. After washing, the secondary antibody was incubated for 1 h at 37°C. Detection step was performed with the Ventana DISCOVERY ChromoMap DAB (ref. 760‐159 Ventana) detection kit. Afterward, the slides were counterstained with hematoxylin II, followed by the bluing reagent (respectively, Cat. no. 790–2208 and 760–2037, Ventana). Sections were then dehydrated, cleared and mounted with permanent mounting and coverslips. Human osteochondral biopsies used as positive control for the specificity of hCol1 staining were collected from leftover surgical material from a patient undergoing unicondylar knee replacement, under the general hospital consent (University Hospital of Basel, Switzerland), following informed consent from the patient, and in accordance with the Ethical Committee of Northwest and Central Switzerland (EKNZ) following Article 2(2)(c) of the Swiss Human Research Act (HRA, RS 810.30). Mouse tibias were used as negative control for hCol1. Images of the histological sections were acquired with a Nikon Ti2 widefield microscope, a Nikon DS‐Ri2 camera and a CFI Plan Apo Lambda NA 0.75, 20X objective. The software used was the NIS‐Elements AR 5.21.03.

### 5.11 QuPath and FIJI Images Quantification

#### 5.11.1 Hematoxylin and Eosin Tissue Classification

Tissue classification into four classes, namely: Bone, Bone marrow, Cartilage and Other was performed as previously described [22, 41] using the open‐source software QuPath v0.3.2. In short, a training image was composed of multiple regions of interest from representative scans to account for tissue variability and annotations for each class were manually drawn. First, tissue was classified into two regions: Cartilage/Bone area and Bone marrow/Fibrotic tissue area. Each of this region was further classified into single region annotations, using parameters: Pixel_classifier_type: “OpenCVPixelClassifier”, Resolution: moderate: (2.93 µm/px), Channels selected (Red, Green, Blue); Scale: 1.0. Features used: “gaussian”, “weighted_std_dev”, “gradient magnitude”, “laplacian”, “structure_tensor_eigenvalue_max”. Results were inspected by an expert and manually corrected if needed. Corresponding training images and classifiers can be found on Zenodo (https://zenodo.org/records/10680128).

#### 5.11.2 Solidity Measurements on Safranin‐O‐Stained Images

Solidity is a quantitative measure of how dense or “solid” an object is. Specifically, it is defined as the ratio of the area of an object to the area of its convex. A solidity value of 1 indicates a perfectly solid object with no holes or irregular boundaries (e.g., a filled circle), while values less than 1 indicate the presence of holes, indentations, or an irregular (non‐convex) shape. In this study, we used the solidity score as a metric for the quality of fusion in between cartilage organoids. Briefly, fused cartilage organoids perimeters were manually detected on safranin‐O‐stained images and the solidity measurements exported using the FIJI software (ImageJ2 Version 2.16.0/1.54p).

#### 5.11.3 Human Collagen 1 Quantification

Representative Pa‐TEC of the 12 weeks (*N = 5*) and the 24 week (*N = 8*) in vivo time‐point from at least 3 independent donors were selected for the quantification of hCol1. Briefly, on the whole section, bone tissue negative for hCol1 was labelled as mouse bone whereas bone tissue positive for hCol1 was labelled human bone. If needed, Hematoxylin and Eosin‐stained images from the same Pa‐TEC were used as reference to help detect bone tissue. In addition, bone detected on the outer part of the sample was labeled *Outer* whereas bone detected in the inner part of the sample was labeled *Core* (Figure S9B).

#### 5.11.4 Human and Mouse Nuclei Detection

Representative Pa‐TEC for each time point (4w, 12 and 24w in vivo) were selected (*N = 3‐5* per time point, at least 3 independent donors). Next, 10 regions of interest (ROIs) 300 × 300 µm were drawn, 5 on the periphery of the sample (Outer) and 5 on the inner part of the sample (Core) on huNu stained tissue sections. For each ROI, human nuclei (brown) and mouse nuclei (blue) were manually counted (Figure S9C).

### 5.12 3‐Point Biomechanical Testing

The mechanical testing of the samples was done using the UMT Tribolab (Bruker, Germany), where force measurements were carried out at a constant, predefined stamp speed of 0.01 mm/s. Custom‐made brackets (span length of 4.5 mm) designed with Solidworks 2022 (Dassault Systems, SolidWorks Corp., France), and 3D‐printed using the P30+ Rapidshape 3D printer (Rapid Shape GmbH 2025, Heimsheim, Germany), were used to handle the Pa‐TEC. Prior to the 3‐point bending tests, supporting brackets were installed on the device to ensure that the loading pin would be lowered exactly in the middle in an axis parallel to the supporting brackets. The calibration was done according to the user manual with a 20 N force cell. Next, Pa‐TEC were placed onto the supporting brackets and the loading pin was manually lowered on the Pa‐TEC center until a measured preload (F_z_) of 0.05N is recorded. To initiate the 3‐point bending test, a preliminary loading step of 15 s designed to ensure firm contact between the loading pin and the Pa‐TEC with an initial force 0.1 ± 0.02 N. Next, the loading pin was lowered at a constant speed of 0.01 mm per second throughout the testing phase while the force (F_z_) was recorded. The tests were stopped according to the following criteria: (i) the tested sample exhibited brittle fracture as indicated by a rapid decrease in the force F_z_ or (ii) tested sample is deformed to the point it loses contact with the supporting brackets. After testing, displacement of the loading pin and measured forces were exported for analysis.

### 5.13 Elastic Modulus Calculations

Based on the 3‐point bending test data we used the following equations to determine the Elastic modulus *E*:

(1) $$E=\frac{F*L^{3}}{d*48I\;}\;$$

(2) $$\varepsilon =\frac{12*c*d}{L^{2}}\;$$

(3) $$\sigma =\frac{F*L*c}{4I}\;$$

*E* is the Elastic modulus, *ε* is the strain and *σ* is the stress. The applied force *F* and the displacement of the loading pin *d* were measured by the UMT Tribolab. The span length *L* = 4.5 mm was determined by the test setup and the distance from the cross‐sectional center of mass *c = 1/2* D sample diameter D was calculated based on the µCT measurements of individual samples, which equaled to half of the average sample diameter *D*.

To calculate the area moment of inertia *(I)*, the following equation was used with the assumption that the cross‐sections of the Pa‐TEC were circular:

(4) $$I=\frac{\pi *D^{4}}{64}$$

After transforming the formulas for the Elastic modulus *E* (Equation 1) and strain (Equation 2) to account for the slope coefficient *r = F / d* [N/mm] of the force‐diplacement curve and sample diameter *(D)*, the following equations were used for the calculations of the mechanical properties:

(5) $$E=\frac{L^{3}}{48I}*r$$

(6) $$\varepsilon =\frac{6*D*d}{L^{2}}$$

After testing all samples, the measured forces *F* and the associated displacement *d* were exported. The examination of the experimental data revealed that a strain range between 0.2%–0.4% for the Pa‐TEC and 0.1%–0.2% for the mouse tibia consistently aligned with the linear region, as a result the elastic modulus *E* was determined in this range.

### 5.14 Statistical Analysis

All statistical analyses were conducted using the software GraphPad Prism version 10.4.1. Differences between groups were analyzed with a one‐way ANOVA, followed by Tukey's post hoc test for multiple comparisons when appropriate. Unpaired comparisons were analyzed using the unpaired t test with Welch's correction and Mann Whitney test. Statistical significances (*p* < 0.05) were reported on the corresponding graphs with the *p* value unless stated otherwise.

## Conflicts of Interest

The authors declare no conflict of interest.

## Supporting information




**Supporting file**: adhm70678‐sup‐0001‐SuppMat.docx

## Data Availability

The data that support the findings of this study are available from the corresponding author upon reasonable request.
