# Supplementary material for: Start, Stop, Rewind, Repeat—Cyclic Exposure of Adipose Stromal Cells‐derived Cartilage Organoids to Chondrogenic and Proliferative Cues to Achieve Scaled‐up and Customizable Bone Formation by Endochondral Ossification
Source: Adv Healthc Mater. 2026 Jan 9;15(14):e04880. doi: 10.1002/adhm.202504880 (PMC13068359; doi:10.1002/adhm.202504880)
Supplement: Supplementary file 1 — Supporting file: adhm70678‐sup‐0001‐SuppMat.docx [file ADHM-15-0-s001.docx]

Supporting Information

Start, Stop, Rewind, Repeat – Cyclic exposure of adipose stromal cells-derived cartilage organoids to chondrogenic and proliferative cues to achieve scaled-up and customizable bone formation by endochondral ossification

Pablo Pfister^1^, Emilien Lhospice^1^, Andres García-García, Robert Paillaud, Sebastian Jung, Romain Schaller, Elisabeth A. Kappos, Claude Jaquiéry, Tarek Ismail, Dirk J. Schaefer, Michael de Wild, Ivan Martin, Alexandre Kaempfen, Arnaud Scherberich* and Adrien Moya*.

^1^Equally contributed to the study

**
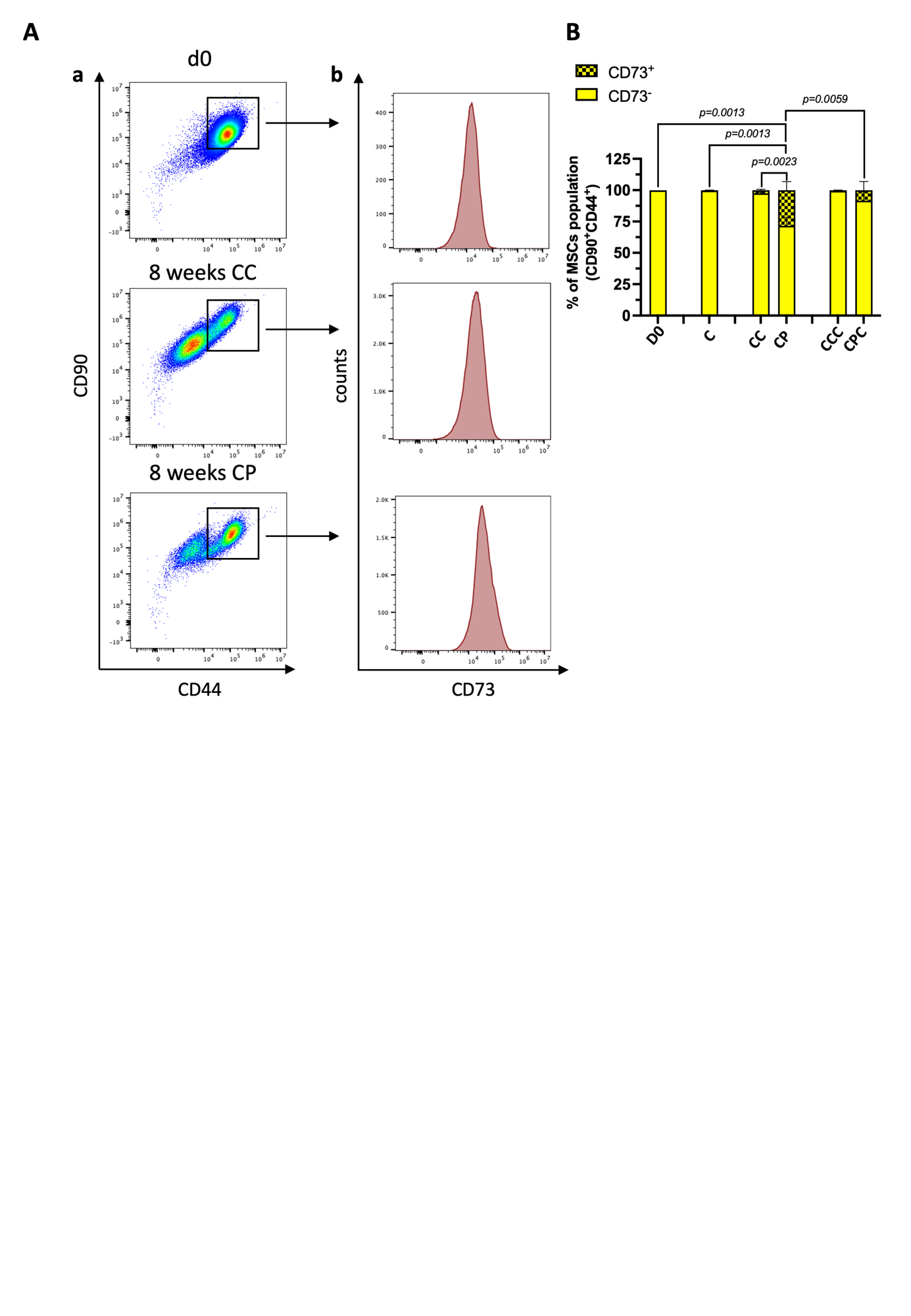
**

**Figure S1. Newly generated MSCs cells are distinct from the starting ASCs P1 population and express the surface marker CD73.** ((**(A)** FACS gating strategy for **(a)** CD90 and CD44 and **(b)** CD73 MSCs fraction (CD90^+^CD44^+^ cells). **(B)** CD73 expression level in MSCs fraction (CD90^+^CD44^+^ cells). data are presented as a percentage (mean ± SD) of the MSCs population. For each time point, 4 cartilage organoids per donor (2 adult donor tested) were digested and analyzed by FACS. For statistical analyses one-way ANOVA with Tukey’s multiple comparisons tests were used.))


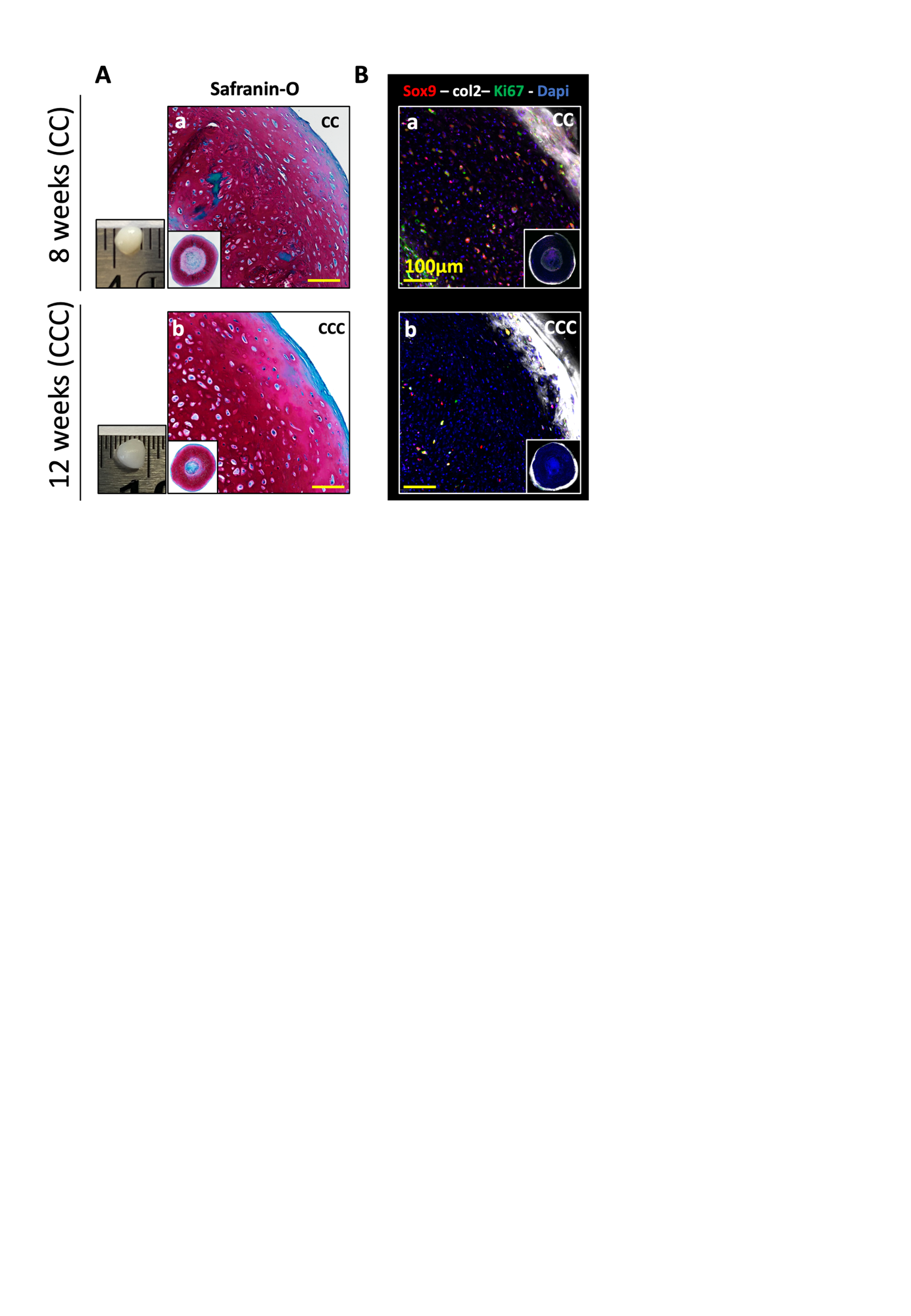


**Figure S2. Evolution of ASCs derived cartilage organoids kept in chondrogenic media for 12 weeks *in vitro***. ((Representative **(A)** Safranin-O staining and **(B)** whole mount images (Sox9, Col2, Ki67) of CT obtained after **(a)** 8 weeks (CC organoid), **(b)** 12 weeks (CCC organoid). (N = 6, 3 biological replicates per donor, 2 adult donors tested). Yellow scale bar = 100µm.))


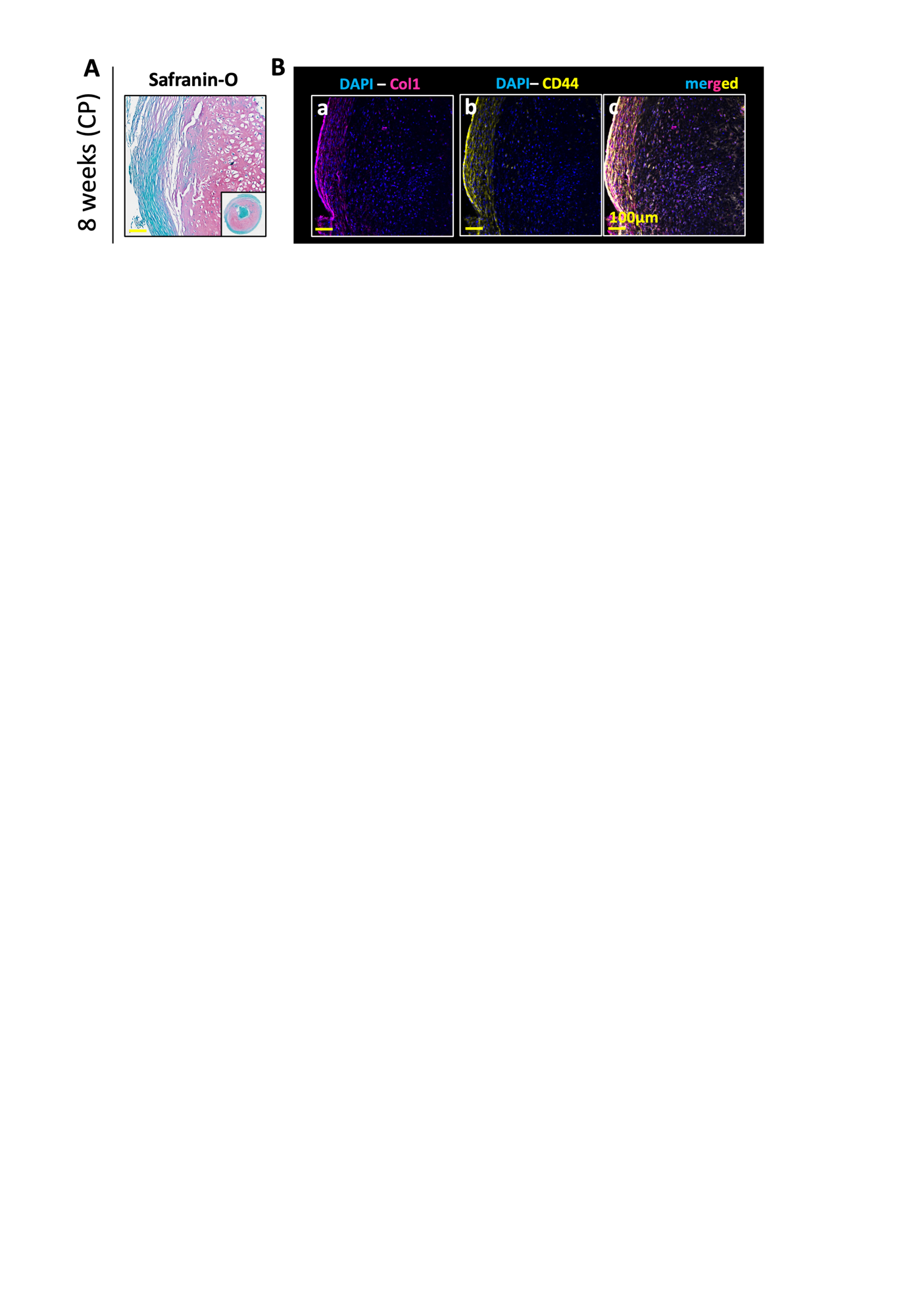


**Figure S3. Newly formed perichondrial cell layer is positive for collagen I and CD44.** ((Representative **(A)** Safranin-O staining and **(B)** whole mount images (Col1, CD44) of cartilage organoids after 8 weeks of culture (CP condition). (N = 3, 3 biological replicates per donor, 1 adult donor tested). Yellow scale bar = 100µm.))


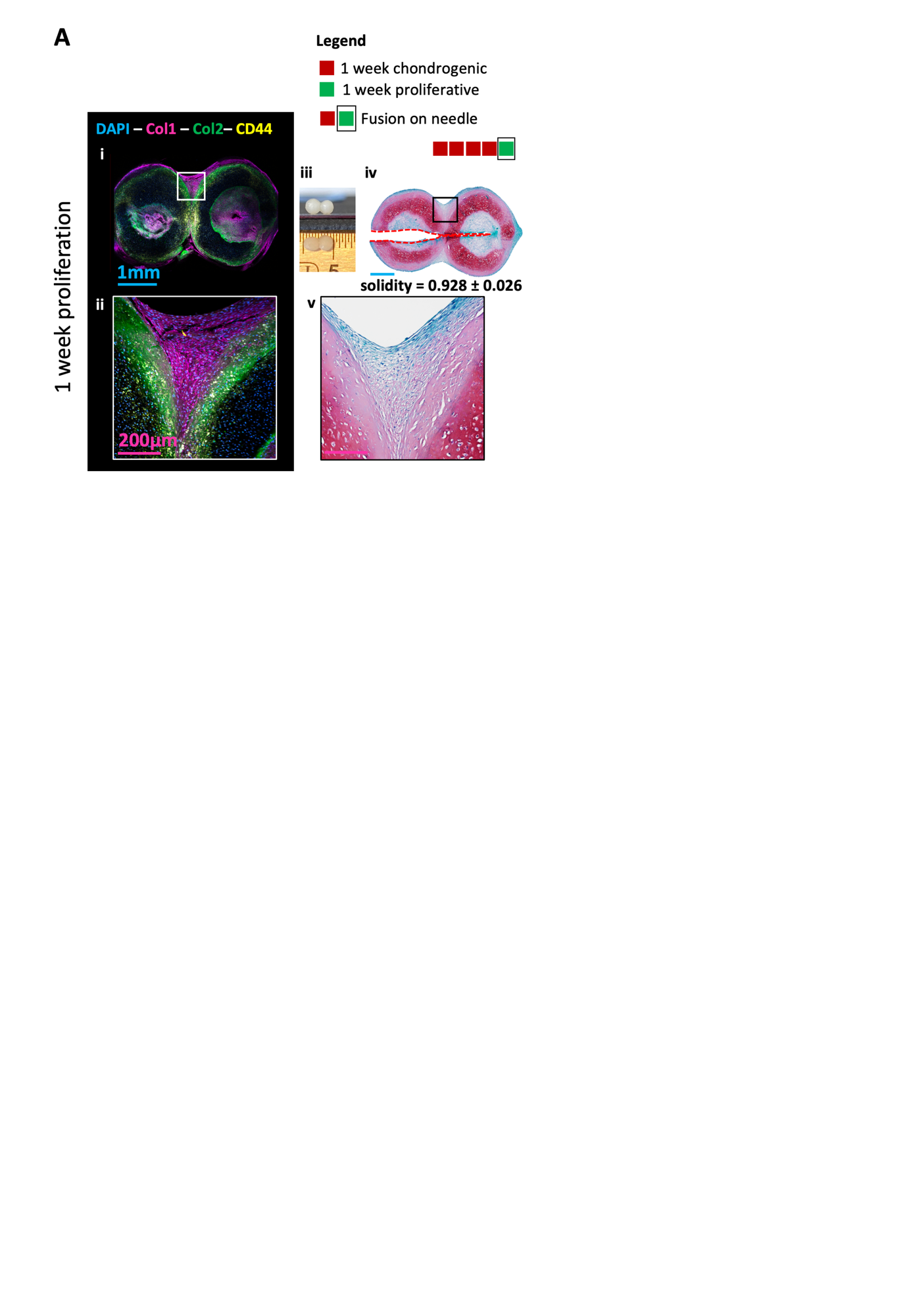


**Figure S4. One week exposure to proliferative cues generates a dense perichondrial layer around and between assembled cartilage organoids.** ((Representative images of **(i-ii)** whole mount staining (Col1, Col2, CD44), **(iii)** macroscopic and **(iv-v)** Safranin-O staining of 4 weeks-cartilage organoids assembled together onto a 27G needle and exposed to proliferative media for 1 week. (N = 3, 3 biological replicates, 1 adult donor tested). Blue scale bar = 1mm and pink scale bar =200µm. Red square indicate 1 week exposure to chondrogenic media, green square indicate 1 week exposure to proliferative media, Black border indicate fusion and cell culture onto the 27G needle. Red dotted lines indicate where the needle was placed.))


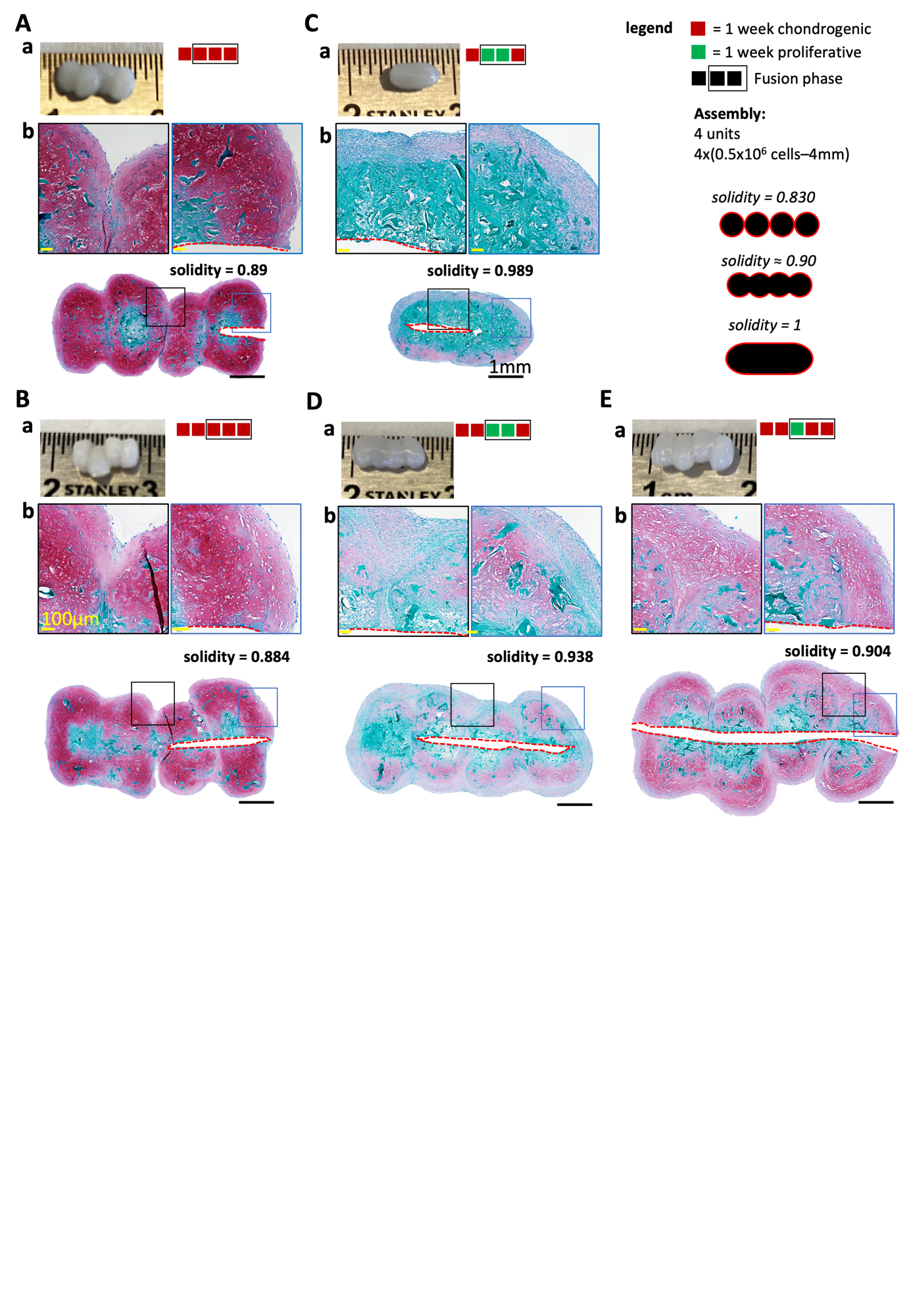


**Figure S5. Impact of proliferative phases on cartilage fusion and maturation.** ((Generation of upscaled cartilage grafts (longitudinal axis), immature ASCs-derived cartilage organoids (1-2 weeks chondrogenic induction) are assembled onto a 27G needle and exposed to **(A,B)** chondrogenic media for 3 weeks, **(E)** 1-week proliferative phase and 2 weeks chondrogenic media or **(C,D)** 2 weeks proliferative media and 1-week chondrogenic media. Representative **(a)** macroscopic and **(b)** Safranin-O-stained images of upscaled cartilage tissues obtained. Black scale bar = 1mm and yellow scale bar =100µm. Red square indicate 1 week exposure to chondrogenic media, green square indicate 1 week exposure to proliferative media, black hollow square indicate fusion onto the 27G needle. Red dotted lines indicate where the needle was placed. Red dotted lines indicate where the needle was placed.))

**
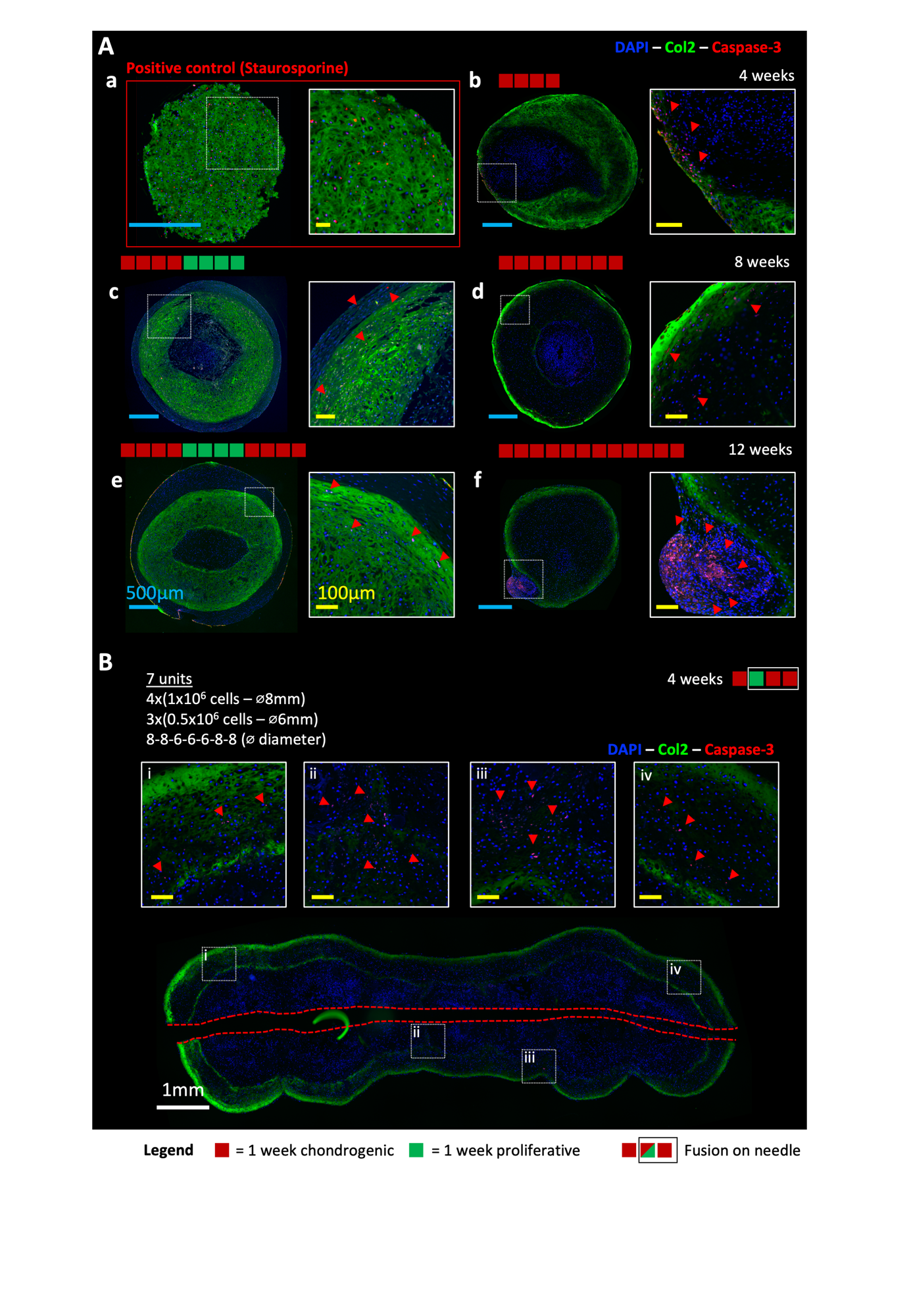
**

**Figure S6. Evaluation of cell death in ASCs derived cartilage organoids.** ((Representative immunofluorescent images stained for (DAPI, Col2, Caspase-3) of **(A)** single cartilage organoids or **(B)** upscaled cartilage grafts (longitudinal axis) comprised of 7 organoids. **(a)** Positive control, cartilage organoid treated with staurosporine 0.1µM for 24 hours to induce cell death. Cartilage organoids cultured for up to 12 weeks in either **(b,d,f)** chondrogenic media only or **(c,e)** with a 4 week proliferative phase. (B) Minimal cell death is observed in upscaled cartilage graft both **(i, iv)** within the cartilage shell or **(ii, iii)** within the undifferentiated core. Red triangles indicate areas were caspase-3 is detected. White scale bar = 1mm, Blue scale bar =500µm and Yellow scale bar =100µm. Red square indicate 1 week exposure to chondrogenic media, green square indicate 1 week exposure to proliferative media, black hollow square indicate fusion onto the 27G needle. Red dotted lines indicate where the needle was placed. Red dotted lines indicate where the needle was placed.))


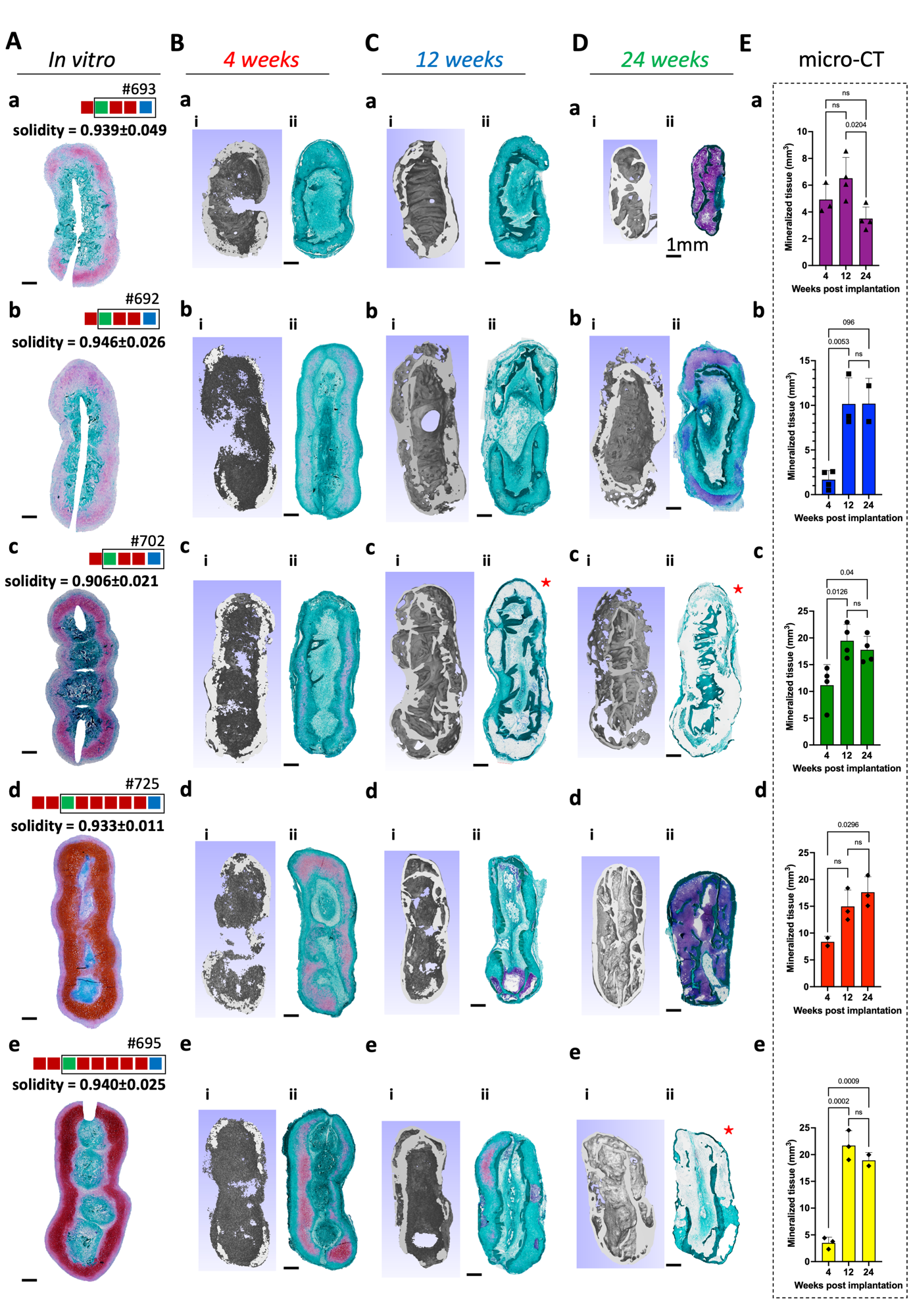


**Figure S7. Individual endochondral ossification for adult donors tested.** ((Pa-TEC (4 cartilage organoids) are generated from adult ASCs p1 (30-56 years old, 1 male donor, 4 female donors) using **(a-c)** a 5-week or **(d-e)** a 9-week protocol—depending on the chondrogenic potential of the donor—before implantation in an ectopic nude mice model. **(A)** Representative Safranin-O-stained images of the Pa-TEC in vitro. Evolution of the bone remodeling in vivo after **(B)** 4 weeks, **(C)** 12 weeks and **(D)** 24 weeks. Representative **(i)** µCT 3D reconstruction and **(ii)** Safranin-O images after implantation in vivo. Evolution of **(E)** mineralized tissue (mm^3^) obtained by μCT after 4-, 12- and 24-week post-implantation. µCT data are expressed in mm^3^ of mineralized tissue (mean±SD). (2-4 biological replicates per donor time point). For statistical analyses one-way ANOVA with Tukey’s multiple comparisons tests were used (ns, p>0.05). Black scale bar = 1mm. Red square indicate 1 week exposure to chondrogenic media, green square indicate 1 week exposure to proliferative media, Black border indicate fusion and cell culture onto the 27G needle, Blue square indicate 1 week exposure to DMEM media. Red star (**⋆**) indicates a biomechanically tested sample resulting in a destroyed bone marrow compartment histologically. For statistical analyses one-way ANOVA with Tukey’s multiple comparisons tests were used (ns, p>0.05).))

**
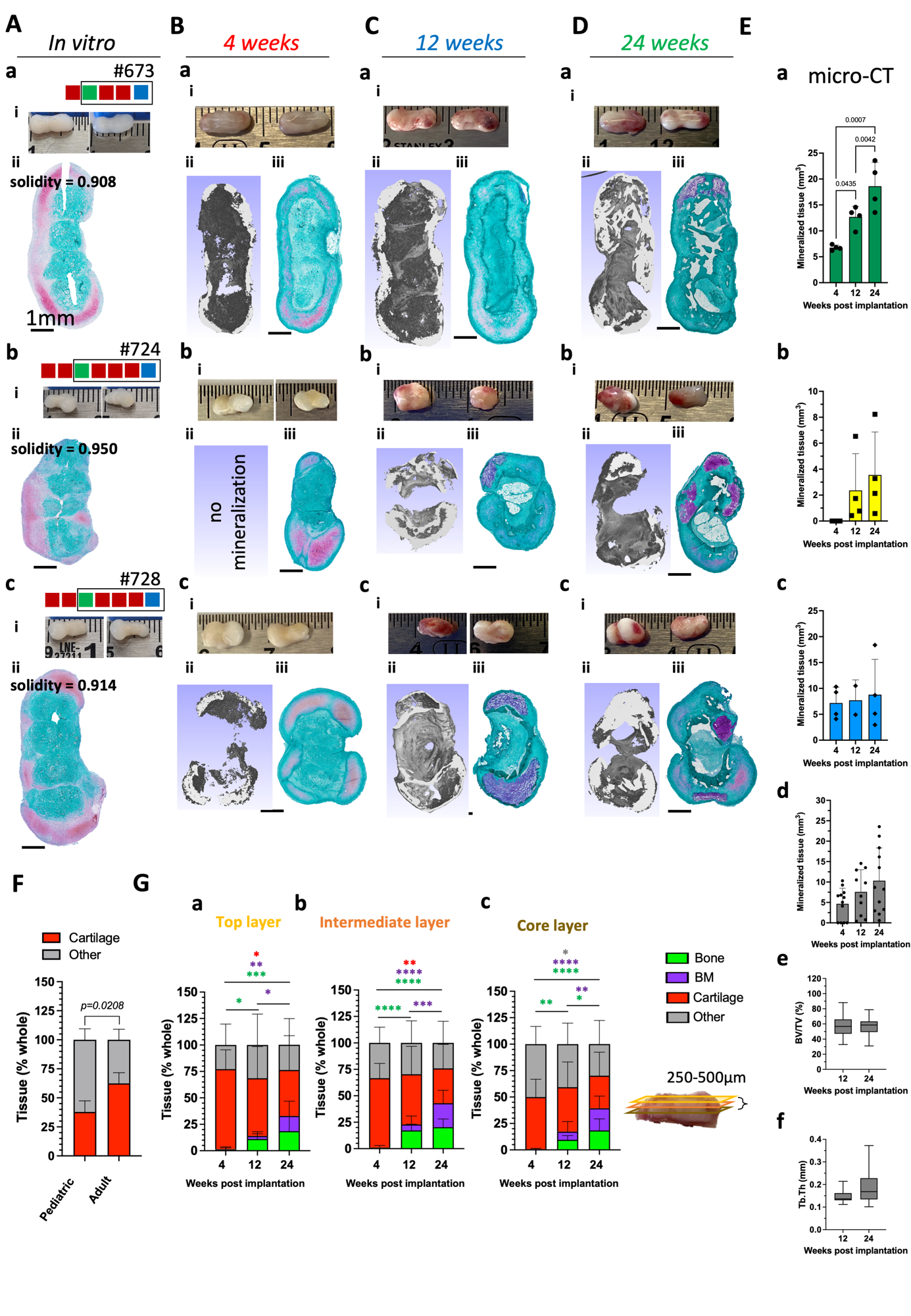
**

**Figure S8. Individual endochondral ossification for pediatric donors tested.** ((Pa-TEC (4 cartilage organoids) are generated from pediatric ASCs p1 (12 -25-month-old donors, 3 male donors) are generated using **(a)** a 5-week or **(b-c)** a 7-week protocol—depending on the chondrogenic potential of the donor—before implantation in an ectopic nude mice model. **(A)** Representative **(i)** macroscopic and **(ii)** Safranin-O-stained images of the upscaled cartilage in vitro. Evolution of the bone remodeling in vivo after **(B)** 4 weeks, **(C)** 12 weeks and **(D)** 24 weeks. Representative **(i)** macroscopic images, **(ii)** µCT 3D reconstruction and **(iii)** Safranin-O images after implantation in vivo. Evolution of **(E)** **(a-d)** mineralized tissue (mm^3^), **(e)** bone volume / total volume (BV/TV in %) and **(f)** trabecular thickness (Tb.Th in mm) obtained by μCT in vivo after up to 24 weeks. Panel (a-c) represent each individual donor and panel (d-f) all the donor combined. Data are expressed as (mean±SD). (N≥10, 2-4 biological replicates per donor, per time point, 3 pediatric donor tested). For statistical analyses one-way ANOVA with Tukey’s multiple comparisons tests was used (ns, p>0.05) for the mineralized tissue (mm^3^) quantification and Mann Whitney test on BV/TV and Tb.Th measurements (ns, p>0.05). **(F)** In vitro cartilage tissue quantification on Safranin-O images. Data are expressed as a percentage (mean ±SD) of the whole tissue (N=6, 2 biological replicates per donor, 3 pediatric donors tested) and compared to the one obtained from adult Pa-TEC (N=5, 1 biological replicates per donor, 5 adult donors tested). Unpaired t test with Welch’s correction were used for statistical analysis. Pediatric Pa-TEC remodel into bone organ similarly to adult Pa-TEC albeit at a slower pace **(G)** **(a-c)** Bone, Bone Marrow, Cartilage and Other tissues quantification within the H&E sections at 3 different depth **(a)** Top Layer, **(b)** Intermediate layer and **(c)** Core layer after 4, 12 and 24 weeks in vivo. On panel **(G)** the statistical significances are represented with stars rather than the p values for clarity. (*p<0.05), (**p<0.01), (***p<0.001), (****p<0.0001). Legend: red square indicate 1 week exposure to chondrogenic media, green square indicate 1 week exposure to proliferative media, Black border indicate fusion and cell culture onto the 27G needle, blue square indicate 1 week exposure to DMEM media. Black scale bar = 1mm.))


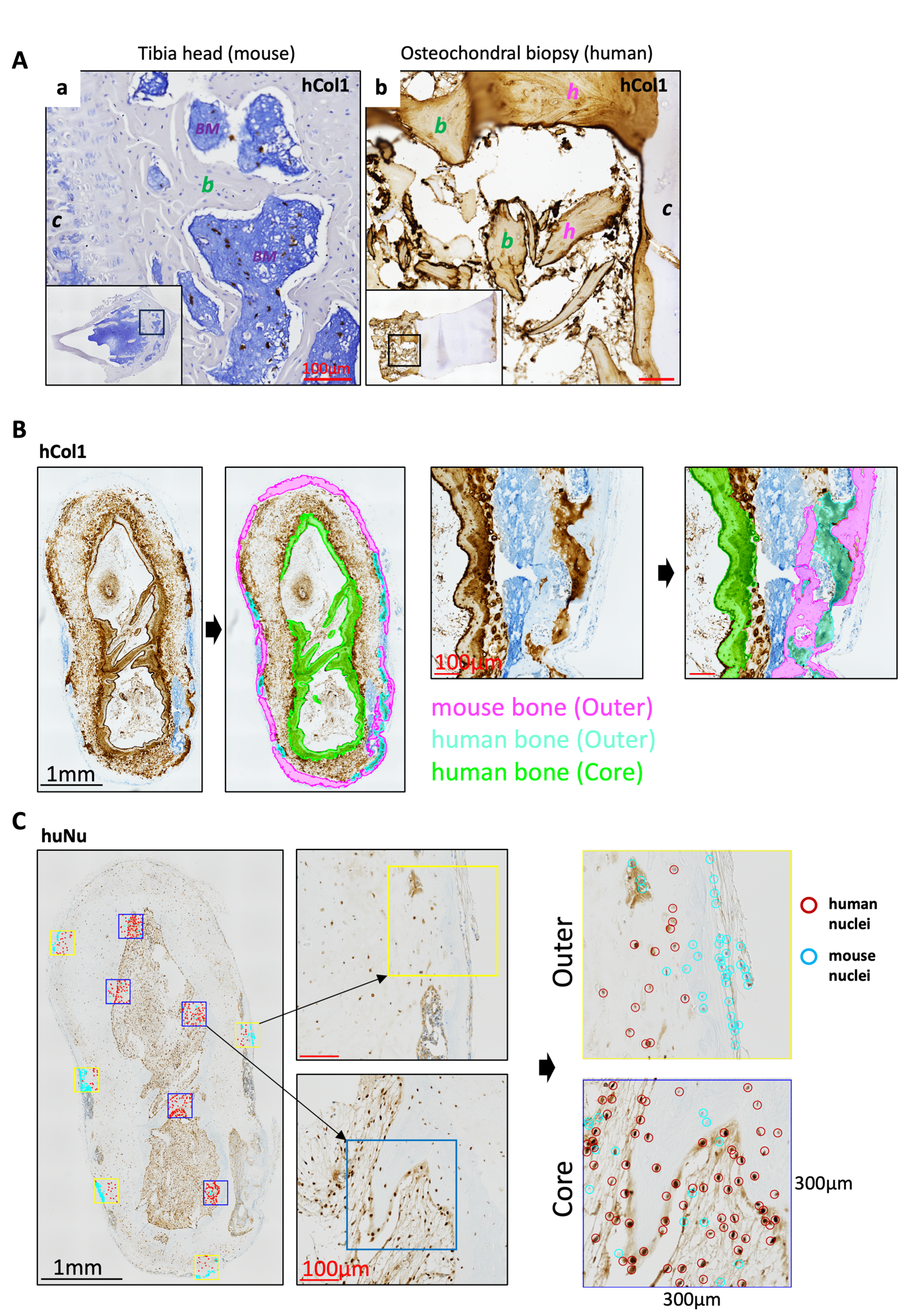


**Figure S9. Immuno-histochemistry supplementary information.** ((**(A)** human Collagen type I (hCol1) staining on **(a)** mouse tibia head (negative control) and **(b)** human osteochondral biopsy (positive control, brown color indicate signal). **(B)** Example of quantification performed on QuPath of **(B)** hCol1 and human nuclei **(C)** from a 12 weeks in vivo implanted Pa-TEC. For human nuclei (huNu) quantification, region of interest (ROI) of 300µm x 300µm are defined either on the outer (yellow, n=5) or inner (blue, n=5) of the Pa-TEC. Mouse nuclei appear blue, huNu appear brown. Bone marrow pockets were excluded from the analysis due to the high density of mouse nuclei. Black scale bar = 1mm, Red scale bar = 100µm. Symbols, cartilage (c), bone (b), bone marrow (BM), human (h).))
